# Virome of Grapevine Germplasm from the Anapa Ampelographic Collection (Russia)

**DOI:** 10.3390/v14061314

**Published:** 2022-06-15

**Authors:** Darya Shvets, Elena Porotikova, Kirill Sandomirsky, Svetlana Vinogradova

**Affiliations:** Institute of Bioengineering, Research Center of Biotechnology of the Russian Academy of Sciences, Leninsky, Prospect 33, 119071 Moscow, Russia; darya-shv@mail.ru (D.S.); plantvirus@mail.ru (E.P.); kirsand@list.ru (K.S.)

**Keywords:** high-throughput sequencing, total RNA, virome, grapevine, germplasm, Anapa Ampelographic Collection, validation, novel virus

## Abstract

Grapevine germplasm collections are unique repositories of grape cultivars; therefore, it is necessary to minimize their infection with pathogens, including viruses, and develop various programs to maintain them in a virus-free state. In our study, we examined the virome of the largest Russian grapevine germplasm collection, the Anapa Ampelographic Collection, using high-throughput sequencing of total RNAs. As a result of bioinformatics analysis and validation of its results by reverse transcription PCR (RT-PCR) and quantitative RT-PCR (RT-qPCR), we identified 20 viruses and 3 viroids in 47 libraries. All samples were infected with 2 to 12 viruses and viroids, including those that cause economically significant diseases: leafroll, fleck, and rugose wood complex. For the first time in Russia, we detected Grapevine virus B (GVB), Grapevine virus F (GVF), Grapevine asteroid mosaic-associated virus (GAMaV), Grapevine Red Globe virus (GRGV), Grapevine satellite virus (GV-Sat), Grapevine virga-like virus (GVLV), Grapevine-associated jivivirus 1 (GaJV-1) and Vitis cryptic virus (VCV). A new putative representative of the genus *Umbravirus* with the provisional name Grapevine umbra-like virus (GULV) was also identified in Russian grape samples.

## 1. Introduction

The grapevine (*Vitis* spp.) is one of the most widely grown and economically important fruit crops in the world. Like most perennial plants, grapevines are affected by a large number of pathogens, including viruses that belong to different taxonomic groups, due to their accumulation during long-term cultivation [1,2,3]. Most often, grapevine viruses are spread by vectors, vegetative propagation through plant cuttings and germplasm [4]. Therefore, the use of virus-free germplasm from the early breeding stages is of particular importance.

For perennial woody plants, germplasm is often maintained in ex-situ field collections [5,6]. Living collections are a valuable source of genetic variability of grape and consist of a large number of samples originating from different geographic regions and representing a range of genetic backgrounds [6,7]. Such plantations maintain modern commercial cultivars, historical cultivars, breeding material, landraces and wild relatives; therefore, the germplasm collections are one of the important sources of grapevine varieties. Grapevine germplasm collections preserve mother vines, from which material is then obtained for sale to commercial vineyards [8].

Preserving the biodiversity of the grapevine is a paramount and urgent task throughout the world. Therefore, many countries maintain germplasm collections to ensure the long-term preservation of grape genetic resources [9]. Germplasm accessions are maintained in repositories located in European countries [10,11], the USA [12], Mexico [13], and Australia [14]. Collections are also grown in the Caucasus, Central Asia, and Mediterranean basin [15,16,17].

According to the Portal of Bioresource Collections [18], there are four collections of grapevine germplasm in Russia: Anapa Ampelographic Collection; Ampelographic Collection “Magarach”; Ampelographic Collection of Daghestan Experimental Station of the N.I. Vavilov Institute of Plant Genetic Resources; and Don Ampelographic Collection of the Ya.I. Potapenko All-Russian Research Institute of Viticulture and Winemaking. The Anapa Ampelographic Collection located at the Anapa Zonal Experimental Station of Viticulture and Winemaking is the largest repository of grapevine germplasm in Russia [19]. It contains varieties imported from various countries of Europe, Asia, America, and regions of Russia. It contains 4921 grape varieties, including *Vitis vinifera* L. (2975), *V. amurensis* Rupr. (40), *V. labrusca* L. (50), interspecies varieties (378), blended interspecies hybrids (290), and other samples. The collection is used for extensive research work on grape cultivars for production and breeding purposes, so it is very important to maintain the germplasm collection in a virus-free state.

According to Fuchs (2020) [3], about 80 grapevine viruses are currently known, however, their effects on the grapevine can be different. Viruses capable of inducing four major diseases lead to serious economic consequences: infectious degeneration and decline (Grapevine fanleaf virus (GFLV), Arabis mosaic virus (ArMV)); grapevine leafroll disease and associated closteroviruses (pathogens under the general name Grapevine leafroll—associated viruses (GLRaVs)); rugose wood (Grapevine virus A (GVA), Grapevine virus B (GVB) and Grapevine virus D (GVD)); and fleck disease (Grapevine fleck virus (GFkV)) [20]. The impact of major viral diseases on the grapevine leads to a decrease in the vitality and longevity of the plant, yield and quality of fruits [2,20]. At the same time, the economic impact on grapevines can be assessed for only a fraction of all detected viruses due to the lack of data on the association of pathogens with symptoms or the latent course of infection [21]. Therefore, it is important to study new viral species and strains that have the potential to cause serious economic losses. The germplasm collection is a potential source of such viruses.

Currently, various methods are used in the diagnostics of grapevine viral infections [22,23]. The health status of propagated material can be assessed by standard detection methods such as PCR-based diagnostic tools and immunoassays (ELISA) which can only detect known and characterized viruses [24]. With the discovery and wide use of high-throughput sequencing (HTS) technology, it became possible to simultaneously obtain information about all harmful organisms (viruses and viroids) present in a plant sample. The detection and characterization of known as well as new, previously undescribed pathogens are among the most successful applications of this technology [25,26,27,28].

In Russia, the distribution of grapevine viruses on commercial, small farms, and abandoned plantations has been studied in the main grape growing regions: the Krasnodar Krai, Stavropol Krai, and the Republic of Crimea. Previously, we have discovered the following economically important viruses and studied their spread: Grapevine rupestris stem pitting-associated virus (GRSPaV), GVA, GLRaV-1, GLRaV-2, GLRaV-3, GFLV, and GFkV [29,30,31,32,33,34]. Using metagenomics methods, we have studied the virome of the vineyards of the Krasnodar Krai and identified: Grapevine Pinot gris virus (GPGV), GLRaV-4, Grapevine Syrah Virus 1 (GSyV-1), Raspberry bushy dwarf virus (RBDV), Australian grapevine viroid (AGVd), and Grapevine yellow speckle viroid 2 (GYSVd-2) [35,36]. At the same time, the diagnostics of viruses and viroids in the plant material of the Russian grapevine germplasm has not been previously carried out.

Since germplasm is used for further breeding and commercial purposes, information is needed on the phytosanitary status of these vineyards. Therefore, the aim of our study was to analyze the virome and study the genetic diversity of viruses in the Anapa Ampelographic Collection in the Krasnodar Krai of Russia. In this work, we used the mRNA-Sequencing method and performed further validation of the results by reverse transcription PCR (RT-PCR) and quantitative RT-PCR (RT-qPCR). This allowed us to detect viruses previously discovered in Russia: GRSPaV, GPGV, GVA, Grapevine virus T (GVT), GFkV, GLRaV-1, -2, -3, -4, Grapevine rupestris vein feathering virus (GRVFV), as well as viroids: Grapevine yellow speckle viroid (GYSVd-1), Hop stunt viroid (HSVd), and AGVd. Moreover, for the first time in Russia, we identified GVB, Grapevine virus F (GVF), Grapevine asteroid mosaic-associated virus (GAMaV), Grapevine Red Globe virus (GRGV), Grapevine satellite virus (GV-Sat), Grapevine virga-like virus (GVLV), Grapevine-associated jivivirus 1 (GaJV-1), and Vitis cryptic virus (VCV). We also report the discovery of a fragment of the genome of a new virus tentatively named Grapevine umbra-like virus (GULV).

## 2. Materials and Methods

### 2.1. Sample Collection and Library Preparation

Phytosanitary monitoring of the grapevine germplasm collection was carried out on the territory of the Anapa Zonal Experimental Station of Viticulture and Winemaking, Krasnodar Krai, in September 2018 and 2019. A total of 47 plants with symptoms of viral diseases were selected for analysis (Supplementary Table S1). Total RNA was isolated from a sample of shoots and leaves for each sample according to the method of Morante-Carriel et al. (2014) [37]. The quality of the isolated RNA was verified using a BioSpectrometer kinetic spectrophotometer (Eppendorf, Hamburg, Germany) and electrophoresis in 1.2% agarose gel. To eliminate DNA, total RNA was treated with DNase I (Thermo Fisher Scientific, Waltham, MA, USA); ribosomal RNA was removed using the RiboMinus Plant Kit (Thermo Fisher Scientific, Waltham, MA, USA) according to the manufacturer’s protocol. RNA concentration was measured on a Qubit 3.0 fluorometer (Invitrogen, Waltham, MA, USA) using the Qubit^®^ RNA BR Assay Kit (Thermo Fisher Scientific, Waltham, MA, USA). To prepare libraries for HTS, we used the NEBNext^®^ Ultra™ II DNA Library Prep Kit for Illumina (New England Biolabs, Ipswich, MA, USA). The quality of the prepared libraries was verified using a Qsep1 capillary electrophoresis system (BiOptic, Taiwan), the concentration was measured on a Qubit 3.0 fluorometer using the Qubit^®^ DNA HS Assay Kit (Thermo Fisher Scientific, Waltham, MA, USA). The 47 obtained libraries were paired-end sequenced (2 × 150 bp) on a NovaSeq 6000 Sequencing System (Illumina, San Diego, CA, USA). FASTQ raw data were deposited at the SRA and are accessible under accession number PRJNA846715.

### 2.2. Bioinformatics Analysis and Virus Identification

The HTS data was processed and analyzed using the Geneious Prime v. 2020.0.4 software package (Biomatters, Auckland, New Zealand) [38]. The resulting raw reads were trimmed with the BBDuk Trimmer tool, the deduplicated reads were paired and merged. For the de novo assembly, we used the SPAdes and Geneious assemblers. The resulting contigs were analyzed using tblastx against the NCBI database of reference viral genomes (uploaded on 21 July 2021). Contigs with plant viruses were counted with E-Value below −40, while contigs with viroids with *E*-Value below −20. Preprocessed in parallel mode reads were mapped using the Geneious Read Mapper with default parameters to the reference genomes of grapevine viruses and viroids. When analyzing the viruses GRSPaV, GVA, GLRaV-1, -2, -4, we also mapped the reads of the library in which they were found to the genome of the nearest isolate. The closest isolate was determined using the blastn analysis of the consensus sequence obtained by mapping the library reads to the reference genome of the corresponding virus. Samples in which the number of reads was more than 10 were considered positive. Nucleotide sequences of the complete genomes of the identified viruses were deposited to the GenBank [39] (Supplementary Table S2).

### 2.3. Phylogenetic and Sequence Diversity Analysi

To determine the nearest isolate using blastn in the NCBI database, we used nucleotide sequences of complete or almost complete genomes (covering more than 90% of the reference sequence) of viruses and viroids.

Phylogenetic analysis included complete or almost complete genomes of Russian isolates found in this study, as well as all world isolates with complete or almost complete (covering more than 90% of the length of the reference sequence) genomes from the GenBank (available as of 15 March 2022). The number of world isolates used in the alignment is shown in Supplementary Table S3. Multiple nucleotide sequence alignments were performed by ClustalW [40] with default parameters in the MEGA X software [41]. Phylogenetic trees were generated using the Neighbor Joining (NJ) method with 1000 bootstrap replicates. The evolutionary distances were calculated using the Maximum Composite Likelihood method. Virus molecular groups were determined based on differences in the sequences of the coat protein gene and complete genomes by clustering on a dendrogram with representative isolates (Supplementary Table S4).

Molecular groups for GLRaV-2 were determined in silico by RFLP analysis according to the method described by us previously [34,35,42].

The pairwise identity (%) of nucleotide and amino acid sequences for representatives of the genus *Umbravirus*, including unclassified *Umbravirus* (umbra-like viruses), was determined with the Sequence Demarcation Tool (SDT v1.2) [43] using the Clustal W alignment algorithm (Supplementary Table S5). Virus ORFs were predicted using the ORFfinder tool [44]. Protein function determination was performed in the InterPro program [45]. Phylogenetic trees were constructed using the Maximum Likelihood (ML) method based on the Tamura-Nei model [46] with 1000 bootstrap replicates.

### 2.4. HTS Data Validation

The results of a bioinformatics analysis were validated by RT-PCR and RT-qPCR. To obtain cDNA, 1 μg of total RNA was mixed with random hexamers and the RevertAid H Minus Reverse Transcriptase (Thermo Fisher Scientific, Waltham, MA, USA) according to the manufacturer’s protocol. As a control for the reverse transcription reaction, we chose the 18S rRNA gene and performed its amplification with primers 18S-H325 (AAACGGCTACCACATCCAAG) and 18S-C997 (GCGGAGTCCTAAAAGCAACA) [47]. For library validation, we used both previously published primers and primers that we designed based on the generated reads and contigs (Supplementary Table S6). RT-PCR was performed using 0.375 U Taq polymerase (Thermo Fisher Scientific, Waltham, MA, USA), Taq Buffer with (NH_4_)_2_SO_4_, 0.2 mM each dNTP, 1 µM each primer and 2.5 mM MgCl_2_.

PCR products were visualized in 1.2% agarose gel and then isolated using the Cleanup Standard kit (Evrogen, Moscow, Russia) according to the manufacturer’s protocol. For each virus, at least one PCR product from one sample was sequenced by the Sanger method using the BigDyeTM Terminator v3.1 Cycle Sequencing Kit (Thermo Fisher Scientific, Waltham, MA, USA) on an ABI PRIZM 3730 automated sequencer according to the manufacturer’s instructions. Sequencing data were analyzed using the Finch TV 1.4.0 software [48] and MEGA X software. The resulting nucleotide sequences were verified by the blastn analysis in the NCBI database. The assembled sequences were deposited to the GenBank (Supplementary Table S7).

HSVd and GYSVd-1 were validated by RT-qPCR using TaqMan^®^ probes (Supplementary Table S8). The main PCR parameters (efficiency, slope, R^2^ and Y-intercept) were determined in a simplex reaction according to the method described by us earlier [49]. RT-qPCR was performed in the BioMaster HS-qPCR reaction mixture (Biolabmix, Novosibirsk, Russia) supplemented with 150 nM each primer, 200 nM probe, and 1 μg of cDNA in triple technical replicates. Amplification was carried out using the LightCycler 96 SW1.1 software (Roche, Mannheim, Germany) at the following conditions: 5 min at 95 °C, followed by 50 cycles of 15 s at 95 °C, 60 s at 60 °C.

## 3. Results and Discussion

### 3.1. Phytosanitary Monitoring of Germplasm Collection

As a result of phytosanitary monitoring in the Anapa germplasm collection, we collected shoots and leaves from different tiers from 47 samples of grapes of various cultivars of Russian and foreign selection (Supplementary Table S1). The samples of germplasm displayed symptoms of four major economically important grape diseases: grapevine leafroll, infectious degeneration, rugose wood, and fleck. The most common symptoms were: leaf deformations, necrosis of the veins, as well as changes in leaf blade coloration of varying degree. A complete list of observed symptoms for each sample is provided in Supplementary Table S1.

### 3.2. Analysis of Sequencing Data of mRNA

Sequencing of mRNA libraries produced an average of 13.3 million reads per library (from 5.2 million reads in the N4 library to 25.7 million reads in the N45 library) (Supplementary Table S9). After preprocessing, the number of reads in most libraries ranged from 1 million to 2 million (from 557,909 for the N4 library to 2,956,466 reads for the N39 library).

As a result of assembly using Geneious, we obtained from 30,922 to 105,732 contigs; N50 ranged from 322 to 466 bp (Supplementary Table S9). The number of contigs obtained as a result of assembly using SPAdes was lower compared to Geneious and ranged from 1480 to 10,944, but the contigs were longer: N50 ranged from 775 to 1462 bp.

The tblastx analysis of contigs obtained using the Geneious assembler identified 17 viruses and 3 viroids, while using SPAdes—14 and 1, respectively (Supplementary Table S10). When analyzing viroid contigs with the *e*-value of −40, we identified only GYSVd-1 (in 34 libraries) and AGVd (in 1 library). No contigs corresponding to HSVd were found, whereas HSVd reads were found in 45 libraries. When the e-value threshold was lowered to −20, HSVd contigs were identified in 46 libraries, as well as GYSVd-1 in another 37 libraries.

As a result of the assembly, 171 complete genomes (with more than 90% coverage of a reference genome) were obtained for 13 viruses and 3 viroids (Supplementary Table S2).

### 3.3. Validation of mRNA Sequencing Results

**Figure 1:**
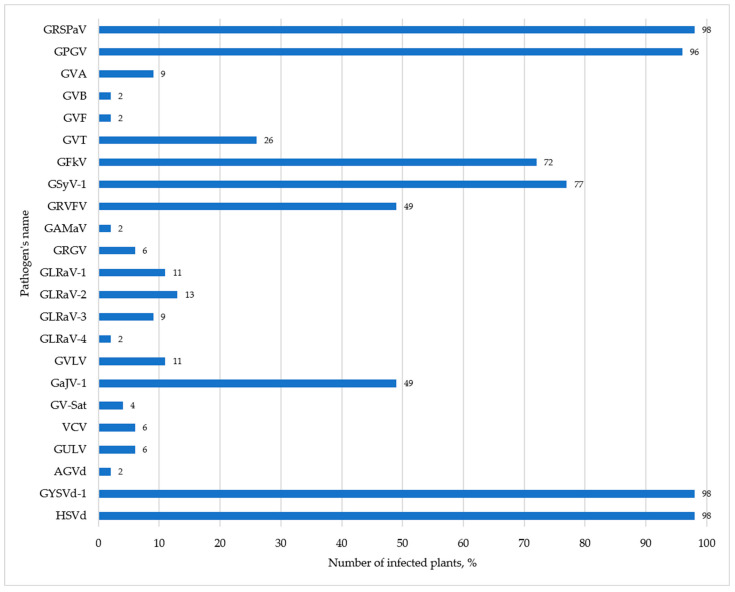
Frequency of distribution of grapevine viruses and viroids in Anapa Ampelographic Collection (percentage of the total number of collected samples).

Validation of 47 libraries confirmed the presence of 20 viruses and 3 viroids in the analyzed samples (Figure 1). The most common (found in more than 50% of libraries) were GRSPaV, GPGV, GFkV, GSyV-1, as well as HSVd and GYSVd-1 viroids, which confirms our previous observations on the wide distribution of these pathogens in Russia [34,35,49]. Not a single virus-free plant was found. All vines had a mixed infection, with the number of viruses per vine ranging from 2 to 12. The most infected sample was 1873 (N28 library) unknown cultivar where we found 10 viruses and 2 viroids, and the least infected sample was 1544 cv. Vanessa Seedless, where we found only GYSVd-1 and HSVd (N11 library).

### 3.4. Family: Betaflexiviridae

**GRSPaV** is a member of the genus *Foveavirus*; it is associated with the Rupestris stem pitting (RSP) disease and probably also with the vein necrosis (VN) disease and Syrah decline disease [50,51,52,53]. However, a direct relationship between the presence of a pathogen and the manifestation of these diseases on the vine is not always observed [54,55]. GRSPaV has been repeatedly noted to be one of the most prevalent viruses in most commercial vineyards in the world [56,57,58]. The presence of GRSPaV has also been found in the grapevine germplasm in Czech Republic [26], Spain [59], Croatia [60], Brazil [61], France [62], Tunisia [63], and Italy [56,64]. Previously, GRSPaV has been found in vineyards in southern Russia [29,34,35].

In our study, based on RNA-seq and the bioinformatics analyses, GRSPaV was detected in 45 libraries (Supplementary Table S10). When mapping the reads of each library to the GRSPaV reference sequence (NC_001948), the complete genome was not assembled for a number of isolates. Therefore, the reads were mapped to the genome of the nearest isolate (Supplementary Table S11). As a result, we assembled 32 complete GRSPaV genomes. The percentage of similarity of Russian isolates at the nucleotide level with the nearest isolates from the GenBank was 83.57–98.75%.

For GRSPaV validation, we used several primer pairs (Supplementary Table S6). With the RScp_F/RScp_R primer pair, the virus was validated in 40 samples. For isolates from the N10, N14, N15, N17, and N34 libraries, the forward primer was replaced in order to amplify the bigger fragment of the GRSPaV coat protein (CP) gene. Based on the results of RT-PCR, we found a product of the expected size in these libraries. In the N2 library, GRSPaV was also detected, although bioinformatics analysis did not find it there. Thus, in our study, GRSPaV was detected in 46 (98%) samples.

On the dendrogram, Russian GRSPaV isolates clustered near representative sequences belonging to different groups. The closest isolates were from Italy, France, Slovakia, USA, Brazil, and China (Supplementary Figure S1a,b). As a result of phylogenetic analysis with high bootstrap support, we found four molecular groups: I, II, III, V. Molecular group V was discovered in Russia for the first time [35]. The Russian group I isolates were quite different from the GRSPaV nucleotide sequences available in the GenBank (identity ranged from 68.9 to 99.9%). In group I, we identified five subclades (a, b, c, d, e, g), each of which, in addition to world isolates, included one or more Russian isolates. Two Russian isolates of the white-berry cultivars Zarif (A1543k) and Kristall (A1560k), which clustered separately, were assigned by us to the subclade g.

In molecular group II, we identified subclades a, b, c. Subclades a and b included the largest number of isolates of this group. The similarity of isolates of group II with isolates from the GenBank at the nucleotide level ranged from 82.08% to 89.65%.

The isolates assigned by us to the genetic group III also exhibited high variability and were distributed over a large number of subclades. The identity of genomes of isolates within group III at the nucleotide level varied from 75.4% to 99.9%. The most different was the isolate from the white cultivar Rkatsiteli (OL961511).

Isolates belonging to group V were the most similar to isolates from the GenBank (identity at the nucleotide level was 97.0–97.4%).

**GVT** is another member of the genus *Foveavirus*, recently identified as a result of transcriptome analysis of the grape [65]. The effect of GVT on the grapevine is still unknown, since it can be present on vines without any visible symptoms [62,66,67]. GVT has been registered in the Slovakia, Czech Republic [68], Germany [69], China [70], Turkey [71], Hungary [67], and Russia [35]. GVT has been detected in the germplasm collections of Croatia [60], France [62], the USA [66], and Italy [64].

As a result of tblastx analysis, we identified contigs corresponding to Panax ginseng flexivirus 1 (PgFV-1) in 10 libraries (Supplementary Table S10). A blastn analysis at the NCBI database showed a high similarity of these contigs to the only closest species—GVT. This result is explained by the absence of the GVT reference sequence in the NCBI’s virus refseq database used for tblastx. Validation of libraries with the previously published GVT-F1/GVT_R primer pair confirmed the presence of GVT in the 10 libraries, as well as in two additional libraries—N7 and N41. Thus, in our study, GVT was detected in 12 samples.

We obtained complete genome sequences of Russian GVT isolates—A1584s, A1873s and A1885s, the similarity of which at the nucleotide level with the closest isolate from the GenBank was 95.89, 95.81, and 89.55%, respectively. According to the results of phylogenetic studies, the identified isolates clustered in two molecular groups: I, IV and V (Supplementary Figure S2) which were also previously found in Russia [35]. The closest were isolates from Italy and France.

**GPGV** is a member of the genus *Trichovirus*; it is found in most viticultural regions of the world [72]. GPGV has previously been found by us in the vineyards of the Krasnodar Krai, Stavropol Krai, and the Republic of Crimea [35,49]. GPGV has also been found to be widely distributed in the germplasm collections of Romania, Ukraine, Bosnia, Montenegro, Serbia, Croatia, Macedonia, Portugal, Spain, Czech Republic, France, Greece, Brazil, and Italy [60,62,64,73,74,75].

In this study, based on RNA-seq and the bioinformatics analyses, we identified GPGV in 43 samples (Supplementary Table S10). GPGV validation was carried out with two primer pairs (Supplementary Table S6). With the GPG-6609F/GPG-7020R primer pair, we identified GPGV in 26 samples, as well as in an additional library—N19. For validation in other libraries where we obtained the GPGV complete genome, we used the second pair of primers, GPgMP_6271_F/GPgMP_6583_R. GPGV was identified in 17 samples, as well as in an additional library—N2. Thus, we identified GPGV in 96% of the analyzed samples.

We assembled 13 complete genomes of GPGV, and the percentage of their similarity with the nearest isolates from the GenBank ranged from 96.92 to 98.54%. The similarity between Russian isolates was 86.54–98.2%. According to phylogenetic analysis, all Russian isolates belong to the same cluster; they cluster together with isolates from France, Italy, Germany, and Slovakia (Supplementary Figure S3).

Grapevine infection with GPGV has previously been found to correlate with the presence of grapevine leaf mottle and deformation disease (GLMD), although many studies have found asymptomatic infection with this virus [25,49,76,77]. It has been shown that a significant role in the manifestation of GLMD symptoms is played by the 3’ region of the MP gene [78], as well as by the synthesis of a shortened (by 6 amino acids) version of the MP protein due to the appearance of an early stop codon as a result of the T/C polymorphism at position 6685 [77]. The alignment of the MP/CP region of the 28 isolates identified in this study with the reference sequence NC_015782 showed that all isolates under study contained C at position 6685, which led to the formation of MP by six amino acids longer than in the reference sequence (Supplementary Figure S4). However, a number of plants were found to exhibit GLMD symptoms, which does not support the existence of a strong correlation of the polymorphism at position 6685 with the presence of symptoms of the disease.

**GVA** is the type species of the genus *Vitivirus*; it is associated with Grapevine kober stem grooving [20]. Its wide distribution in the world has been repeatedly noted [79,80]. In Russia, GVA was previously found in the vineyards of the Krasnodar Krai, Stavropol Krai, and the Republic of Crimea [30,34,35]. There is evidence of its presence in the grapevine germplasm in the USA [81], Spain [59], Croatia [60], Brazil [61], France [62], as well as Italy [64,82].

In this study, GVA was identified in four samples (Supplementary Table S10). The mapping of reads to the genome of the nearest isolate made it possible to increase the completeness of the assembly of the genomes of three isolates (Supplementary Table S11). We assembled the complete nucleotide sequences of four GVA isolates and analyzed them by blastn, which revealed a rather low similarity to the sequences of the nearest isolates from the GenBank, ranging from 81.41 to 85.97%. Pairwise identity of isolates among themselves was 82.15–99.4%.

Phylogenetic analysis showed that all isolates belong to GVA molecular group I (Supplementary Figure S5). The closest were isolates from South Africa. This molecular group is the most widespread in Slovakia, Turkey, Iran, and Tunisia [83,84,85,86]. Moreover, when we examined the coat protein gene of isolates detected in our previous studies in the Krasnodar Krai and the Republic of Crimea, we also identified isolates of GVA molecular groups II and IV [34,35].

**GVB** is a member of the genus *Vitivirus*. It can cause corky bark disorder which is a component of the grapevine rugose wood complex [87,88]. GVB is not a widespread virus, but its ability to affect graft incompatibility makes it a harmful organism, the absence of which must be confirmed in planting material [20,89]. GVB has previously been found in the grapevine germplasm in Italy [82], the USA [81], Brazil [61], Croatia [60] and in the Portuguese National Ampelographic Collection [90].

GVB was bioinformatically identified in the N28 library and then validated by RT-PCR (Supplementary Table S10). Using blastn, was found that the Russian isolate A1873p has a 90.14% similarity at the nucleotide level with an isolate from the USA (JX513897). The isolate we identified belonged to the molecular group II and clustered most closely with the Portuguese isolates (Supplementary Figure S6). Isolates belonging to groups I and II have previously been found in the grapevine germplasm in Portugal [90]. 

**GVF** is a member of the genus *Vitivirus*. It has first been identified in a sample of grapevine cv. Cabernet sauvignon with symptoms of graft incompatibility when propagated on various rootstocks [20,91]. The presence of GVF has been reported in the USA [91], South Africa [92], and Greece [93]. GVF has also been found in the USDA National Clonal Germplasm Repository (NCGR) in California [81] and in the germplasm collections of Croatia [60] and France [62].

In our study, GVF was bioinformatically detected and validated in the N45 library (Supplementary Table S10). Blastn analysis revealed the similarity of the nucleotide sequence of the complete genome of the isolate A1888q with an isolate from South Africa (MW309671) at the level of 92.59%.

On the phylogenetic tree, all GVF sequences available in the GenBank are distributed into two main clusters (Supplementary Figure S7). One of them includes isolates from Greece, Japan, and USA, the other isolates from South Africa and Greece. A Russian isolate with 85% bootstrap clustered next to isolates from South Africa.

**GVH** (Grapevine virus H) is a member of the genus *Vitivirus*; it was first detected in an asymptomatic sample of an unknown grapevine cultivar from Portugal [94]. GVH has been identified in Greece [95], Croatia [96], and in California in collection samples of NCGR [81]. As a result of RNA-seq and the bioinformatics analyses, we identified reads mapping to GVH in three samples (N12, N28, N36). Based on the obtained reads, we designed three primer pairs (Supplementary Table S6), but we were unable to confirm by RT-PCR the presence of GVH in any library. The absence of PCR products may be due to a low titer of GVH in the grapevines.

### 3.5. Family: Tymoviridae

**GFkV** is a member of the genus *Maculavirus*. It causes fleck, which is a ubiquitous disease reported in most of the world’s winemaking countries [97]. The presence of GFkV has been reported in the germplasm collections of Italy [64,82], Czech Republic [26], Spain [59], Brazil [61], France [62], and Croatia [60,98]. In Russia, we have previously found GFkV on the territory of the main grape-growing zones [34,35].

Based on RNA-seq and the bioinformatics analyses, we identified GFkV in 25 libraries (Supplementary Table S10). To validate GFkV, we used two primer pairs (Supplementary Table S6). Using the GFkV 6351F/GFkcp_R primer pair, we were able to confirm the presence of GFkV in 24 samples, as well as in 9 additional libraries (N1, N7, N8, N12, N13, N31, N35, N37, N48). By replacing the forward primer with GFkcp_F, a PCR product of the expected size was obtained for the N9, N14, N26, and N30 libraries. Thus, GFkV was found in 72% of the analyzed samples from the Anapa collection.

We assembled 13 complete GFkV genomes. Using BLASTn analysis, we determined the identity of the obtained nucleotide sequences with the sequences of the closest isolates from the GenBank at the level of 89.79–94.81%. Sequence analysis within the group of Russian isolates showed a fairly high diversity: the level of identity was 82.4–91.7%. All Russian isolates on the dendrogram were distributed into three clusters: A, B, C (Supplementary Figure S8); the A1584c isolate clustered separately.

**GAMaV** belongs to the genus *Marafivirus*; it is associated with grapevine asteroid mosaic disease. Infected leaves of *Vitis vinifera* vines exhibit star-shaped chlorotic spots [20]. The distribution of GAMaV is limited not only in Canada and USA, where it was first discovered [97,99,100], but also in Japan [101], Uruguay [102], France [103], Italy [104], and Spain [105]. To our knowledge, GAMaV has not been detected in the world’s grapevine collections.

Based on RNA-seq and the bioinformatics analyses, GAMaV was detected and validated in the N2 library, where we found one contig assembled from four reads (Supplementary Table S10). Due to a low coverage of the reference sequence (7.8% with a pairwise identity of 94.5%), we were unable to assemble the complete GAMaV genome and perform phylogenetic analysis.

**GRVFV** is a member of the genus *Marafivirus*. It was first discovered in Greece on a grapevine that showed symptoms of vein feathering after graft inoculation on *Vitis rupestris* [106]. It is a quarantine virus for grapevine certification in Australia [107]. Since its first discovery, GRVFV has been reported in most viticultural areas of the world [99,108,109,110,111]. In addition to commercial vineyards, GRVFV has been found in the germplasm collections of New Zealand [112], Spain [59], Brazil [61], Croatia [60], Czech Republic [26], France [62], as well as in the Swiss nuclear stock collection [113]. In Russia, GRVFV was first described in the vineyards of the Krasnodar Krai [35].

GRVFV contigs were found in six libraries—N13, N15, N28, N37, N41, N45 (Supplementary Table S10). Validation with the GRVFV_F/GRVFV_R primer pair confirmed the presence of GRVFV in these libraries, as well as in 17 additional libraries.

**GSyV-1** is a member of the genus *Marafivirus*. It was first identified on Syrah grapevines with severe decline symptoms in the USA [114]. Despite its wide distribution in viticultural regions of the world, data on its effects on the grapevine and interaction with other viruses are still limited [60,97,115,116]. GSyV-1 has been detected in the germplasm collections of Croatia [60], Czech Republic [26], and Brazil [61]. In Russia, this pathogen has been detected in the Krasnodar Krai [35].

Based on RNA-seq and the bioinformatics analyses, GSyV-1 contigs were identified in four libraries—N10, N24, N25 and N46 (Supplementary Table S10). During validation, we found the presence of GSyV-1 in these libraries, as well as in 32 additional samples. Thus, GSyV-1 was found in 77% of the samples we analyzed.

**GRGV** is a member of the genus *Maculavirus*. Together with GAMaV, GRVFV, and GFkV, it constitutes the fleck complex [97]. To date, there are no data on grapevine symptoms associated with GRGV [20]. It is difficult to ascertain symptoms for GRGV due to the presence of other viruses on the analyzed plants, including representatives of the Tymoviridae family [107]. Despite unclear symptomatology, the presence of GRGV has been reported in several European countries, namely Italy, Albania [117], Greece [106], Germany [118], France [119], as well as in Uruguay [102], China [120], the USA [121], and Iran [122]. GRGV has been reported in the germplasm collections of Brazil [61], Spain [107], Croatia [60], and France [62]. GRGV has not been previously detected in Russia.

In this study, GRGV was found in the N24 library, for which we obtained 1 contig and 14 reads (Supplementary Table S10). To validate the results of bioinformatics, we used three primer pairs for amplification of several regions of different lengths from the GRGV genome (Supplementary Table S6). With the first primer pair (GRGV_6083F/GRGV_6386R) selected by us for the only contig from the N24 library, and we were able to confirm the presence of GRGV in this sample, as well as in two additional libraries—N27 and N45. With the second primer pair (RG6061F/RG6801R) published previously for amplification of a 741 bp fragment of the CP gene, we obtained a positive result for the N24 and N45 libraries. With the third primer pair (RG4847F/RG6076R) targeted at amplifying a 1230 bp fragment of the RNA-dependent RNA polymerase (RdRp) gene, we identified GRGV only in the N24 library. The absence of target fragments in other samples may be due to the large size of the PCR product, the nonspecificity of primer annealing to the genomes of Russian GRGV isolates, and the use of a variable gene, RdRp, as a template. However, the use of three primer pairs made it possible to detect GRGV in three samples.

### 3.6. Family: Closteroviridae

Grapevine leafroll disease (GLD) is one of the major economically important diseases affecting the grapevine; it is found in all viticultural regions of the world [20,123]. Infection with GLD-associated Grapevine leafroll-associated viruses (GLRaVs) has been found in various grapevine germplasm collections in Brazil [61], Spain [59,107], Croatia [60,98], the USA [81,114], Italy [64,82], Georgia [124], France [62], Czech Republic [26], Algeria [125], and China [126]. In Russia, GLRaV-1, -2, -3 and -4 have previously been detected in the main grape growing areas by various molecular methods [31,32,33,34,35].

There are six species of Grapevine leafroll-associated viruses: GLRaV-1, GLRaV-3, GLRaV-4, and GLRaV-13 belong to the genus *Ampelovirus*, GLRaV-2 and GLRaV-7 belong to the genera *Closterovirus* and *Velarivirus*, respectively [3]. There often exist multiple strains for each GLRaV species [127].

#### 3.6.1. GLRaV-1

According to the results of RNA-seq and the bioinformatics analyses, GLRaV-1 was detected in five libraries—N16, N23, N30, N45, N48 (Supplementary Table S10). To validate samples, we used two previously published primer pairs. With the GLRaV-1_F/GLRaV-1_R primer pair, we identified the target product in the N16, N30, N45, and N48 libraries. With another primer pair, GLRaV-1_13339F/GLRaV-1_13739R, a PCR product was obtained for N23 library. Thus, GLRaV-1 was confirmed in five grape samples.

As a result of mapping the reads of each library to the GLRaV-1 reference sequence (NC_016509), complete genomes were obtained for 5 isolates. At the same time, the mapping of the reads to the genome of the isolate closest to the one we found made it possible to increase the completeness of the assembly of their genomes (Supplementary Table S11). The blastn analysis of the sequences of the complete genomes of Russian isolates showed similarity with isolates from the GenBank at the level of 86.59–99.65% (Supplementary Table S2). The identity of 3 isolates was less than 90%. The similarity between Russian isolates ranged from 81.37% to 93.4%. Russian isolates clustered most closely with isolates from Poland, China, USA, Nigeria, and South Korea (Supplementary Figure S9a,b). As a result of the analysis of the dendrogram, the isolates were assigned to groups I and II with high bootstrap support. Previously, we have identified only molecular groups II and III of the virus in the southern regions of Russia [34,35].

#### 3.6.2. GLRaV-2

Based on RNA-seq and the bioinformatics analyses, GLRaV-2 was identified in 6 grapevine samples (Supplementary Table S10). In the N10 and N39 libraries, complete genomes were assembled that were identical to the GLRaV-2 reference sequence by 89.6% and 89.8%, respectively; in the N19 library—contigs identical to the reference sequence of one of the strains of GLRaV-2—Grapevine rootstock stem lesion-associated virus (GRSLaV). As a result of mapping the reads of the N19 library to the isolate closest to the isolate we identified, we were able to increase the completeness of the genome assembly from 38.6% to 61.5% (Supplementary Table S11).

In three more libraries (N28, N30, N41), we obtained contigs and reads that mapped to the reference genomes of both GLRaV-2 and GRSLaV with varying degrees of their coverage (Supplementary Table S10). In the N28 library, a complete GLRaV-2 genome was assembled de novo (identity with the reference was 85.4%). In the N30 library, a de novo assembled complete genome was 70.6% identical to NC_004724 GRSLaV, 69.3% identical to NC_007448 GLRaV-2 and 93.4% identical to the closest GLRaV-2 isolate, MH814492. In the N41 library, complete genomes of GLRaV-2 and GRSLaV were assembled de novo; the identity between the nucleotide sequences was 69.9%, which indicates the presence of two strains in one sample. Thus, we assembled six complete genomes of Russian GLRaV-2 isolates, including two genomes of its GRSLaV strain.

The blastn analysis revealed the identity of the nucleotide sequences with the nearest isolates from the GenBank at the level of 99.39–99.53% (Supplementary Table S2). A comparison of the isolates with each other showed that their identity ranged from 69.75% to 99.4%.

To validate GLRaV-2, we used primers V2dcpf2/V2cpr1; RT-PCR confirmed its presence in the N10, N28, N39, and N41 libraries (Supplementary Table S10).

To validate GLRaV-2 in the N19 and N30 libraries, we selected primers GLRaV2_12482F/GLRaV2_12858R for the GLRaV-2 contigs identified in them. As a result, GLRaV-2 was detected in these two libraries.

To confirm the presence of two GLRaV-2 strains in the N41 library, we selected two primer pairs (GLRaV2_15986F/GLRaV2_16490R and GRSLaV _15982F/GRSLaV _16490R) for divergent regions of the coat protein genes (Supplementary Table S6). As a result, we obtained an amplicon from each of them, which was sequenced by the Sanger method (Supplementary Table S7). The sequences obtained using primers for GLRaV-2 were 100% identical to the complete genome of GLRaV-2 assembled de novo, while using primers for GRSLaV—99.9% identical to this strain. Thus, we confirmed the presence of two GLRaV-2 strains in the N41 library.

A phylogenetic analysis of the coat protein gene sequences of Russian and world isolates of GLRaV-2 in our samples revealed the presence of three molecular groups of this virus: H4, PN, and PV20 (Supplementary Figure S10a–d). The closest isolates were from Italy, Spain, France, and Argentina. PV20 has not been previously identified in Russia [34,35]. Isolates A1588j (GRSLaV, N41 library) and A1876e (GLRaV-2, N30 library) with 98% bootstrap support clustered in the same subclade with representative sequences of the PV20 group. Representative isolates of the GLRaV-2RG group formed an additional subclade (Supplementary Figure S10b). Thus, according to our data, GRSLaV and GLRaV-2-RG belong to different molecular groups, although they were previously described as representatives of the same group [128]. The isolates of the H4 group (N28 library) identified by us with 99% bootstrap support clustered next to the Russian isolates that were detected earlier [34,35].

As a result of digestion of fragments of the coat protein gene with TaqI and RsaI restriction endonucleases, we identified both already known groups and those that were not previously described (Supplementary Table S12). The profile obtained by in silico digestion of the CP fragment of the A1873e isolate with TaqI and RsaI coincided with those previously published [34,42] and was assigned by us to group 3.

As a result of restriction digestion of the CP fragments of GLRaV-2 isolates A1543e (N10 library), A1584e (N39), A1588e (N41), we obtained profiles differing from those previously published. Digestion with TaqI produced a profile that coincided with the profile of groups 1a, 1b, and 2, while digestion with RsaI—a profile that coincided with the profile of groups 1b and 2, which did not allow us to accurately identify the group for these isolates [42].

A similar situation occurred with the sequences of isolates of the PV20 group. The profile obtained by digestion of the V2dCPf2/V2dCPrl amplicon of samples A1588j (N41 library) and A1876e (N30) with TaqI was identical to the profiles of groups 3 and 4 [42], with RsaI—to the profile of group 1a. On the phylogenetic tree, these isolates clustered next to representative sequences of the PV20 group, but A1876e with 98% bootstrap support clustered separately (Supplementary Figure S10b). As a result of digestion of one of the representative sequences of this clade (MW715832) with restriction endonucleases TaqI and RsaI, a profile similar to the Russian isolates A1588j (N41 library) and A1876e (N30 library) was obtained (Supplementary Table S12). Therefore, the isolates A1588j and A1876e that we identified, together with the representative isolate MW715832, were assigned to the new group 7.

The results of detection of GLRaV-2 in samples of the ampelographic collection by bioinformatics and molecular biology methods using several primer pairs (some of which were designed to detect the GRSLaV strain) once again confirm the need to combine these methods for the most complete characterization of the plant virome and for further study of the virus.

#### 3.6.3. GLRaV-3

GLRaV-3 was bioinformatically detected in four libraries—N14, N24, N28 and N30. Using the previously published GLRaV-3_F/GLRaV-3_R primers, these data were confirmed by RT-PCR (Supplementary Tables S6 and S10). The blastn analysis of four complete genomes of the isolates we detected showed that the identity with the nearest GenBank isolates at the nucleotide level comprised 99.49–99.59% (Supplementary Table S2). The identity of the genomes of the isolates between themselves ranged from 91.39% to 99.47%. On the dendrogram, the isolates clustered in one clade with representative sequences of groups I and II (Supplementary Figure S11a,b) that we identified in previous studies [34,35]. The closest were isolates from Portugal, the USA, Brazil, and South Africa.

#### 3.6.4. GLRaV-4

Based on RNA-seq and the bioinformatics analyses, we identified in the N22 library contigs and reads corresponding to the reference sequence of GLRaV-4 and its strains GLRaV-5 and GLRaV-6. As a result of mapping the library reads, the coverage of reference sequences comprised 7.4% for GLRaV-4, 97.2% for GLRaV-5 and 33.3% for GLRaV-6 (Supplementary Table S10). At the same time, the reads that mapped to GLRaV-4 and GLRaV-6 also mapped to GLRaV-5. This indicates that only the GLRaV-5 strain was present in the studied sample. As a result of mapping the reads to the nearest sequence MF669481, 99.3% of the reference genome of the GLRaV-5 strain was assembled.

To validate the libraries, we used previously published LR-5-HYF/LR-5-HYR primers; as a result, the presence of the GLRaV-5 strain of GLRaV-4 in the N22 library was confirmed. The identity of this isolate with the closest isolate from the GenBank (MF669481.1) was 93.38%. In the dendrogram, it clustered next to isolates from Brazil and Pakistan (Supplementary Figure S12).

### 3.7. Family: Virgaviridae

**GVLV** (Grapevine virga-like virus) is a poorly studied member of the genus *Tobamovirus* that was first discovered by HTS on a grapevine in Brazil [129]. GVLV has a single-stranded positive sense RNA genome possessing an alpha-like replication complex, which allowed to assign it to the Virgaviridae family, despite its low identity with viruses from this family [3,129]. At the moment, the GVLV genome remains only partially assembled: the sequences of the helicase (Hel) domain (MK257732) and the RNA-dependent RNA polymerase (RdRp) (MK257731) are available in the GenBank. There are reports of other viruses, the genomes of which are similar to the genome of GVLV. For instance, a novel Grapevine associated jivivirus 1 (GaJV-1) has previously been detected in Spanish and Italian samples; the contigs of this virus are identical to flavi-like and virga-like viruses [130]. The GaJV-1 genome is represented by RNA1 (MN520745), RNA2 (MN520746), RNA3 (MN520747).

Our local database includes reference sequences of two GVLV domains and three GaJV-1 segments, to which we mapped the reads of all libraries. In 13 libraries, we identified from 1 to 10 reads corresponding to the RdRp gene of GVLV and in 18 libraries—from 1 to 7 reads corresponding to the Hel gene of GVLV (Supplementary Table S10). In addition, in 23 libraries we identified from 1 to 10 reads corresponding to RNA1 of GaJV-1, in 20 libraries, we found from 1 to 12 reads corresponding to RNA2 of GaJV-1, and in 10 libraries, from 1 to 6 reads corresponding to RNA3 of GaJV-1. 

For validation, we selected primers for the GVLV and GaJV-1 reads. The GvirgaRdRp_1008F/GvirgaRdRp_1520 primer pair designed for RdRp of GVLV allowed us to obtain a PCR product of the expected size in four libraries (N13, N20, N24, N38). The GvirgaHel_1718F/GvirgaHel_2105R primer pair designed for Hel of GVLV allowed us to amplify the target product in five libraries (N12, N16, N22, N24, N41). The amplicons were sequenced by the Sanger method. The blastn analysis of the RdRp nucleotide sequence of a sample from the N24 library showed that its identity with the RdRp gene of a Spanish isolate of GVLV (MK257731; 100% query coverage) was 98.29%, while the identity with RNA2 of an Italian isolate of GaJV-1 (MN520746.1; 100% query coverage) was 97.86%. For three nucleotide sequences of the helicase domain of N12, N24, and N41 samples, the closest genome with a query coverage of 99% was found to be only an Italian isolate of GaJV-1 (MN520745.1); its identity with the three sequences was 98.71%, 97.94% and 98.71%, respectively. Thus, according to blastn analysis, the amplicons obtained by us had identity with both GVLV and GaJV-1. When we used the GaJV-1_480F/GaJV-1_890R primer pair, a PCR product of the expected size was found in 23 samples. Sanger sequencing of the amplicon from the N29 library and its blastn analysis showed a 98.2% identity with GaJV-1.

Thus, for the first time in Russia, we identified recently discovered GVLV and GaJV-1 viruses in a germplasm collection. In our study, the results of bioinformatics analysis and RT-PCR did not always coincide, which may be associated, on the one hand, with a low titer of viruses, and on the other hand, with insufficiently good knowledge of their genomes. Further research is needed to establish the phylogenetic relationships of these viruses in order to more accurately detect them in samples. There is also a need to study the biology of GaJV-1, since its initial detection in grapevine samples infected with oomycete *Plasmopara viticola* does not allow the grape to be definitively named the host of this virus. The inclusion of GaJV-1 in the group of plant viruses requires further study, considering that symptoms of infection by fungal pathogens and oomycetes were found in Russian collection grape cultivars, as well as taking into account its possible relationship with grapevine fungal endophyte [131].

### 3.8. Family: Partitiviridae

**VCV** is a putative member of the genus *Deltapartitivirus* which was recently identified in wild vines of *Vitis coignetiae* [132]. By now, it was found in Japan and China [132,133]. The VCV genome is represented by two-segmented double-stranded (ds) RNAs encoding RdRp and CP [132].

As a result of RNA-seq and the bioinformatics analyses, we did not find VCV contigs, since the reference sequence of this virus is not available in the refseq database of the GenBank. However, we identified contigs of the Sinapis alba cryptic virus 1 (SaCV-1) in the N17, N43, N46 libraries and the Pepper cryptic virus 1 (PCV-1) in the N44 library, which belong to the same genus as VCV. The blastn analysis confirmed that these contigs correspond to VCV.

Mapping the reads to RNA1 of VCV (LC602838) showed that it was present in the N17, N42, N43, N44, and N46 libraries. In the N17, N43, and N46 libraries, the percentage of RNA1 assembly was more than 92. Mapping the reads to RNA2 of VCV (LC602839) showed that it was present in the N17, N43, and N46 libraries. Complete RNA2 sequences were obtained from the N43 and N46 libraries (Supplementary Table S10). The identity of the Russian sequences for RNA1 of VCV with the isolates available in the GenBank was from 95.01% to 95.97%, for RNA2 from 90.64% to 92.91%. In the dendrogram built for the CP and RdRp genes, Russian isolates clustered into a separate clade (Supplementary Figure S13a,b).

To validate the results of bioinformatics analysis, we selected primers for the CP gene of the VCV genome (Supplementary Table S6). A PCR product of the expected size was obtained in three libraries (N17, N43, N46). Thus, we for the first time identified this virus in collection samples in Russia.

### 3.9. Family: Unassigned

**GV-Sat** is a member of the genus *Virtovirus*. It has first been discovered in California in the vineyards of the Davis Grapevine Virus Collection (DGVC) and the USDA National Clonal Germplasm Repository (NCGR) [134]. GV-Sat has also been found on the cultivar Askeri in the INRA collection in France [135], in the ampelographic collection in Slovenia [136,137] and in the vineyards of Hungary [138]. GV-Sat has not been previously detected on the territory of Russia.

In our study, we identified GV-Sat in two libraries, N16 and N48, which was validated by RT-PCR with the GV-Sat_433/GV-Sat_876 primers (Supplementary Tables S6 and S10). Al Rwahnih et al. (2013) suggested a possible role of *Vitivirus* and Grapevine Leafroll-associated virus species in the replication of GV-Sat [134]. Czotter et al. (2018) detected GVA and GLRaV-1 in samples infected with GV-Sat, while Miljanić et al. (2021) detected only GLRaV-1 and GLRaV-2 [136,138]. In the N16 and N48 libraries, we also detected GVA and GLRaV-1, which confirms the hypothesis of their possible helper function for GV-Sat.

We assembled two complete GV-Sat sequences. The identity of isolates A1562m and A1892m at the nucleotide level with the nearest isolate from the GenBank was 95.53% and 94.81%, respectively, while their identity between themselves was 96.9%. In the dendrogram, the detected GV-Sat isolates clustered in two clades together with isolates from USA (Supplementary Figure S14).

### 3.10. Family: Tombusviridae

#### Identification of Putative New Species from the Genus *Umbravirus*

Based on RNA-seq and the bioinformatics analyses, we identified in the N44 library one contig of 649 bp in size using the of Geneious and SPAdes assemblers; it had homology with the nucleotide sequence of Carrot mottle mimic virus (CMoMV), the type member of the genus *Umbravirus* of the Tombusviridae family. The blastn analysis of the nucleotide sequence of the contig (with the megablast option) found no identity with any virus in the NCBI database. The blast analysis with the function “Somewhat similar sequences” revealed different levels of identity with representatives of the family Tombusviridae (genera *Umbravirus* and *Alphanecrovirus*), as well as the family Totiviridae (unclassified Totiviridae). The highest identity was observed with the nucleotide sequence of an isolate of the Strawberry virus A (StrVA, MK211274) from the genus *Umbravirus* (identity of 68.98%; 51% query coverage corresponding to the putative replicase domain). The blastp analysis with 216 aa of the predicted RdRp sequence of representatives of the genus *Umbravirus* showed that the closest species are Wheat umbra-like virus (WULV) (identity with isolate UIN24849.1 is 71.36, 98% query cover) and StrVA (identity with isolate QGX02202 is 66.20; 100% query coverage). Based on the low identity of the identified sequence with the genomes of viruses of the genus *Umbravirus* available in the database, we made an assumption about the presence of a novel virus named Grapevine umbra-like virus (GULV).

**Figure 2:**
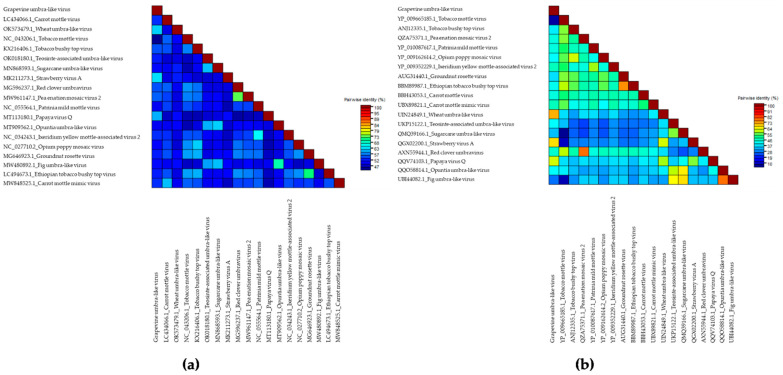
Color-coded pairwise identity matrix performed for the Russian isolate of the Grapevine umbra-like virus and representatives of the genus *Umbravirus* for (**a**) nucleotide sequences, (**b**) amino acid sequences of RNA-dependent RNA polymerase (RdRp) gene. GenBank accession numbers for each isolate are presented in Supplementary Table S5.

We performed a comparison of the nucleotide sequences of the RdRp gene of GULV and 18 representatives of the genus *Umbravirus* and umbra-like viruses. The identity ranged from 62.6% for StrVA to 44.0% for Tobacco mottle virus (TMV) (Figure 2a, Supplementary Table S13). Moreover, a high identity was found with two other species—Wheat umbra-like virus (WULV) (61.5%) and Papaya virus Q (PpVQ) (59.7%). The identity matrix performed for amino acid sequences showed a wide range of values from 70.8 to 6.5%. The maximum pairwise identity was obtained with WULV (70.8%), PpVQ (60.9%) и StrVA (66.2%) (Figure 2b; Supplementary Table S14).

**Figure 3:**
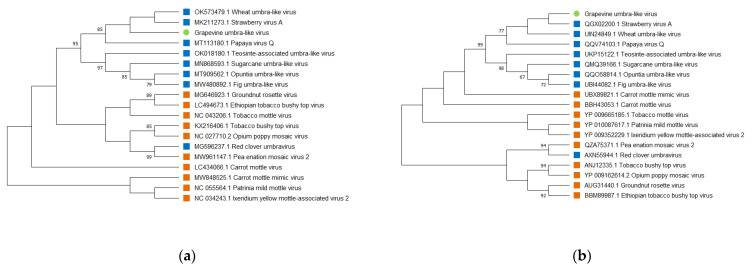
Phylogenetic analysis of (**a**) nucleotide sequences, (**b**) amino acids sequences of RNA-dependent RNA polymerase (RdRp) gene of a Russian isolate of Grapevine umbra-like virus (marked with a green label), representatives of the genus *Umbravirus* (marked with an orange label) and unclassified *Umbravirus* (marked with a blue label). The tree was constructed using the Maximum Likelihood method. The numbers at the nodes indicate bootstrap values >60% (1000 replicates). GenBank accession numbers for each isolate are presented in Supplementary Table S5.

The results of pairwise comparison of the nucleotide and amino acid sequences of GULV and representatives of the genus *Umbravirus* were confirmed by phylogenetic analysis. In the dendrogram built for the nucleotide sequences of isolates of umbraviruses, GULV clustered near StrVA and WULV (Figure 3a). PpVQ was also grouped in this clade with 85% bootstrap support. In the dendrogram built for the RdRp amino acid sequences, GULV, StrVA, WULV, and PpVQ clustered with 77% bootstrap support into a separate clade (Figure 3b). Moreover, phylogenetic trees showed that GULV, along with most other unclassified *Umbravirus*, formed a separate clade. 

To validate the identified GULV contig, we selected the GULV_227F/GULV_506R primer pair for this contig. As a result of PCR, we obtained a 280 bp amplicon in the N31, N37 and N44 libraries. At the same time, the reads of other libraries were not mapping to the GULV contig. The obtained amplicon was sequenced by the Sanger method and showed an identity at the level of 99.5% with the sequence of the contig obtained using HTS.

When plants are infected with viruses from the genus *Umbravirus*, symptoms manifest themselves as mottles or mosaics of leaves [139]. In our study, sample 1872 (N37 library), in which the GULV sequence was identified, did not manifest symptoms of viral infections, while two other samples, 1883 and 1886, where the GULV sequences were identified, had symptoms characteristic of grapevine infection with economically significant viruses: leafroll, marbling, double knots on vines. GULV was found in samples along with GRSPaV, GFkV, GSyV-1, GaJV-1, GYSVd-1, HSVd, GRVFV, GPGV, GLRaV-2, which makes it impossible to distinguish the symptoms caused by this particular virus. Moreover, the absence of novel symptoms characteristic of GULV in plants can be explained by the absence of a possible virus-associated satellite RNA that is known to affect the symptomatology of viruses from the genus *Umbravirus* [139,140].

As demarcation criteria for assigning a species to the genus *Umbravirus*, nucleotide sequence identity of less than 70% and natural host range are used [141]. In our work, we, for the first time, identified a partial nucleotide sequence of RdRp of a novel virus that had an identity of 62.6% with StrVA. It should be noted that none of the previously known viruses of the genus *Umbravirus* has the grape as a plant host. Despite the fact that we assembled only a part of the genome of the new virus, its close relationship with representatives of *Umbravirus* and umbra-like viruses and the fulfillment of two criteria allowed us to suggest that this is a possible new member of umbra-like viruses in the Tombusviridae family, and we gave it a provisional name: Grapevine umbra-like virus (GULV). The assembly of the complete GULV genome and its annotation, the detection of possible helper viruses and the determination of symptomatology will be the subject of future research.

### 3.11. Family: Pospiviroidae

Viroids, among them the Grapevine yellow speckle viroid 1 (GYSVd-1) and Australian grapevine viroid (AGVd) from the genus *Apscaviroid* and the Hop stunt viroid (HSVd) from the genus *Hostuviroid* are the most common infectious agents of the grapevine (*Vitis* spp.) [142,143]. Usually viroids do not cause symptoms, with the exception of GYSVd-1 and -2 which are the causative agents of Yellow speckle (YS) of the grapevine and in combination with GFLV can cause Vein-banding (VB) disease [20,21].

GYSVd-1 and HSVd are distributed worldwide [144,145]. These viroids were detected in the germplasm collections of China [146], Spain [59,107], Czech Republic [26], Italy [64,144], and the USA [114]. In vineyards of the Krasnodar Krai, GYSVd-1 and HSVd were found in more than 50% of infected samples [35].

HSVd contigs were identified in 43 libraries. As a result of mapping the reads of each library to the reference sequence, HSVd was found in 46 libraries (Supplementary Table S10). RT-qPCR revealed the presence of HSVd in 46 (98%) samples. The main parameters of RT-qPCR are shown in Supplementary Table S8. The results of RT-qPCR were confirmed by RT-PCR of samples with the HSVd-78P/HSVd-83V primers followed by Sanger sequencing of the resulting amplicons (Supplementary Table S7).

We assembled 43 complete HSVd genome sequences. The identity between Russian isolates was at the level of 94.3–100%, while the identity with isolates from the GenBank was from 99.6% to 100% (Supplementary Table S2). Phylogenetic analysis showed that 39 isolates clustered near isolates of the Hop group (Supplementary Figure S15a–f), the host plants for most of which were *Vitis Vinifera* [147]. A total of 11 isolates clustered with representative sequences from the Plum-Hop/cit3 group, host plants for most of which were stone fruits. The HSVd isolates identified by us in previous studies also belonged to the Hop group and Plum-Hop/cit3 group [35].

Based on RNA-seq and the bioinformatics analyses, we identified GYSVd-1 contigs in 37 libraries and assembled 38 complete genomes (Supplementary Table S10). GYSVd-1 was detected by RT-qPCR in 42 samples, as well as in four additional libraries (N1, N17, N20, N23). The main parameters of RT-qPCR are shown in Supplementary Table S8. RT-PCR with the GYSVd-1-mF/GYSVd-1-mR2 primers confirmed the presence of GYSVd-1 in grapevine samples (Supplementary Table S7). Thus, all samples that we analyzed were infected with GYSVd-1.

In the dendrogram, Russian GYSVd-1 isolates were distributed uniformly, which indicates their genetic diversity. The identity of isolates between themselves ranged from 75% to 100%, while with the nearest GenBank isolates from 95.64% to 100%. The most different was the isolate from cv. Zarif (A1543b). Russian isolates clustered closest to isolates from Croatia, Czech Republic, USA, Slovakia, Brazil, China, South Korea, and Slovenia (Supplementary Figure S16a–f).

Unlike HSVd and GYSVd-1, which are characterized by their widespread presence, AGVd is found only sporadically [148]; its presence has been reported in the vineyards of Iran [149], Tunisia [150], India [151], Turkey [152], Australia [153], the USA [114], Thailand [142], China [154], Greece [155], and Russia [35]. In addition, AGVd was found in the Grapevine Germplasm Resources Garden in Beijing [146] and the germplasm collections in Italy [144]; to our knowledge, these are the only records of the presence of this viroid in grapevine collections.

In our study, AGVd was detected using bioinformatics analysis and RT-PCR in one sample of the N7 library (Supplementary Table S10). Based on blastn analysis, the closest isolate with 93.96% identity was the isolate from India (MH476217). Given the low heterogeneity of AGVd, it is currently unknown if different molecular groups of the viroid exist [144]; however, on the phylogenetic tree, several clusters can be distinguished (Supplementary Figure S17) that are mainly divided by geography. The Russian isolate closely clustered with isolates from China and Iran.

Thus, HTS of total RNA from samples of the Anapa germplasm collection allowed us to expand the available information on the genetic diversity of the identified viruses in Russia. Bioinformatics analysis based on de novo assembly of viral, as well as mapping the reads to a local database which included all the reference sequences of viruses that infect grapes, allowed us to most fully characterize the plant virome. Using two assemblers, Geneious and SPAdes, we were able to obtain contigs and assemble 171 complete genomes for 13 viruses and 3 viroids. For the first time in Russia, we detected the presence of GVB, GVF, GAMaV, GRGV, GV-Sat, GVLV, GaJV-1, and VCV. The Anapa collection was found to be free from GFLV and ArMV, whereas representatives of other viruses causing the economically important diseases were identified in grapevine samples: GLRaV-1, GLRaV-2, GLRaV-3, GLRaV-4, GVA, and GFkV. We also assembled one contig of a putative umbra-like virus and gave it a provisional name: Grapevine umbra-like virus (GULV).

The results of bioinformatics analysis were validated by RT-PCR or RT-qPCR. However, due to low sequencing depth and/or low concentration and uneven distribution of viruses in infected samples, it was not always possible to validate HTS results. As a result, the number of positive validation results exceeded the expected value for some viruses. The use of several pairs of primers for the detection of each virus made it possible to study the viral load in a particular plant in more detail.

Multiple infections can have a more serious impact on a plant than infection with a single virus [156]. In total, we identified 23 viruses and viroids in the collection; in 6 of them, the prevalence rate exceeded 70%. Not a single virus-free plant was found; all plants were infected with several viruses or strains of one virus. Such a high viral load can negatively affect the existence of the collection and further use of the grapevines.

To prevent further spread of pathogens in plant material, various infection control methods can be used. To obtain virus-free planting material or to decontaminate infected plants, in vitro methods are often used [157]. A common practice in the sanitation of grape samples is the use of meristem culture and its combination with thermotherapy, chemotherapy and somatic embryogenesis [158]. The use of advanced agricultural technologies can help reduce the number of vectors, one of the main ways of spreading viruses in the plantation. In addition, the use of certification systems allows to prevent the planting of seedlings infected with the main dangerous grapevine viruses. According to the FAO/IPGRI Technical Guidelines, germplasm must be obtained only from material tested for pathogens [159]. Viral pathogens are especially dangerous because of the asymptomatic course of the infection. There exist protocols regulating the testing of plant material for the presence of certain grapevine viruses and viroids [24,160]. GFLV, GLRaV-1, GRLaV-3, and GFkV can negatively affect the assessment of phenotypic traits, therefore, they are paramount for testing in plant germplasm material [24].

Today in Russia there are no documents and regulations governing the presence and qualitative composition of viral pathogens of grapes both on imported material and on existing viticultural plantations. However, the existing program “Development of viticulture, including nurseries” is aimed at the use of virus-free planting material at new plantations. For this purpose, it is necessary to develop modern molecular genetic methods of diagnostics of pathogens and pests of grapes and to conduct testing for grape phytopathogens of viral, bacterial, and fungal etiology by molecular methods.

## 4. Conclusions

The combination of visual inspection and modern molecular diagnostics methods, such as high-throughput sequencing of total RNA and validation by RT-PCR and RT-qPCR, made it possible to determine the phytosanitary status of the Anapa Ampelographic Collection. The wide variety of detected pathogens confirms the need for screening in other collections and regions of Russia. Obtaining data on phytosanitary monitoring will reduce the risk of further spread of viruses to new vineyards. Moreover, more information on the presence and genetic diversity of viruses is required for the development of integrated grape protection, as well as for the development of prophylactic programs and systems of planting material certification. The biological value of plant material of germplasm must be maintained at a high level for future selection research and production programs.

## Data Availability

Representative sequences were deposited in GenBank under the accession numbers: ON548147-ON548162, ON548898, ON567239-ON567255, ON584178-ON584187, ON620217-ON620256, ON645908-ON645921, ON669137-ON669253, ON703249-ON703251, ON711252-ON711253.

## Supplementary Materials

The following supporting information can be downloaded at: https://www.mdpi.com/article/10.3390/v14061314/s1, Figure S1: Phylogenetic tree showing the distribution of coat protein nucleotide sequences of Russian Grapevine rupestris stem pitting-associated virus isolates; Figure S2: Phylogenetic tree showing the distribution of complete genome nucleotide sequences of Russian Grapevine virus T isolates; Figure S3: Phylogenetic tree showing the distribution of complete genome nucleotide sequences of Russian Grapevine Pinot gris virus isolates; Figure S4: Alignment of MP/CP nucleotide sequences and movement proteins (MP) of Russian Grapevine Pinot gris virus (GPGV) isolates; Figure S5: Phylogenetic tree showing the distribution of complete genome nucleotide sequences of Russian Grapevine virus A isolates; Figure S6: Phylogenetic tree showing the distribution of coat protein nucleotide sequences of Russian Grapevine virus B isolate; Figure S7: Phylogenetic tree showing the distribution of complete genome nucleotide sequences of Russian Grapevine virus F isolate; Figure S8: Phylogenetic tree showing the distribution of complete genome nucleotide sequences of Russian Grapevine fleck virus isolates; Figure S9: Phylogenetic tree showing the distribution of coat protein nucleotide sequences of Russian Grapevine leafroll-associated virus 1 isolates; Figure S10: Phylogenetic tree showing the distribution of coat protein nucleotide sequences of Russian Grapevine leafroll-associated virus 2 isolates; Figure S11: Phylogenetic tree showing the distribution of complete genome nucleotide sequences of Russian Grapevine leafroll-associated virus 3 isolates; Figure S12: Phylogenetic tree showing the distribution of complete genome nucleotide sequences of Russian Grapevine leafroll-associated virus 4 isolates; Figure S13: Phylogenetic tree showing the distribution of complete genome nucleotide sequences of Russian Vitis cryptic virus (VCV) isolates; Figure S14: Phylogenetic tree showing the distribution of complete genome nucleotide sequences of Russian Grapevine satellite virus isolates; Figure S15: Phylogenetic tree showing the distribution of complete genome nucleotide sequences of Russian Hop stunt viroid isolates; Figure S16: Phylogenetic tree showing the distribution of complete genome nucleotide sequences of Russian Grapevine yellow speckle viroid 1 isolates; Figure S17: Phylogenetic tree showing the distribution of complete genome nucleotide sequences of Russian Australian grapevine viroid isolate. Supplementary Tables: Table S1: Basic information of sampled vineyards and viral infection; Table S2: Complete sequences of viruses and viroids uploaded into GenBank with their identifier; Table S3: Initial data complete or almost complete genomes for phylogenetic analysis; Table S4: Representative virus isolates used for group definition; Table S5: List representative umbraviruses and umbra-like viruses; Table S6: List of the RT-PCR primers used for virus detection; Table S7: List of sequences after Sanger sequencing submitted to the GenBank; Table S8: List of the RT-qPCR primers used for virus detection and calibration curve parameters for simplex RT-qPCR; Table S9: Number of reads and contigs obtained for each library after sequencing by Illumina NovaSeq 6000 Sequencing System; Table S10: Bioinformatics analysis and its validation; Table S11: Bioinformatics analysis: mapping of preprocessed reads to closest genome; Table S12: RFLP patterns of the GLRaV-2 coat protein gene amplicons; Table S13: The pairwise identity matrix of RdRp gene nucleotide sequences of GULV and 18 representatives of the genus *Umbravirus* and umbra-like viruses; Table S14: The pairwise identity matrix of RdRp gene amino acid sequences of GULV and 18 representatives of the genus *Umbravirus* and umbra-like viruses.
