# Supplementary material for: Virome of Grapevine Germplasm from the Anapa Ampelographic Collection (Russia)
Source: Viruses. 2022 Jun 15;14(6):1314. doi: 10.3390/v14061314 (PMC9230720; doi:10.3390/v14061314)
Supplement: Supplementary file 1 [file viruses-14-01314-s001.zip › Supplementary Figures.pdf]

Virome of Grapevine Germplasm from the Anapa Ampelographic Collection (Russia)

Darya Shvets, Elena Porotikova, Kirill Sandomirsky and Svetlana Vinogradova *

Institute of Bioengineering, Research Center of Biotechnology of the Russian Academy of Sciences, Leninsky Prospect 33, 119071 Moscow, Russia; darya-shv@mail.ru (D.S.); plantvirus@mail.ru (E.P.); kirsand@list.ru (K.S.)
*Correspondence: coatprotein@bk.ru (S.V.)

Supplementary materials

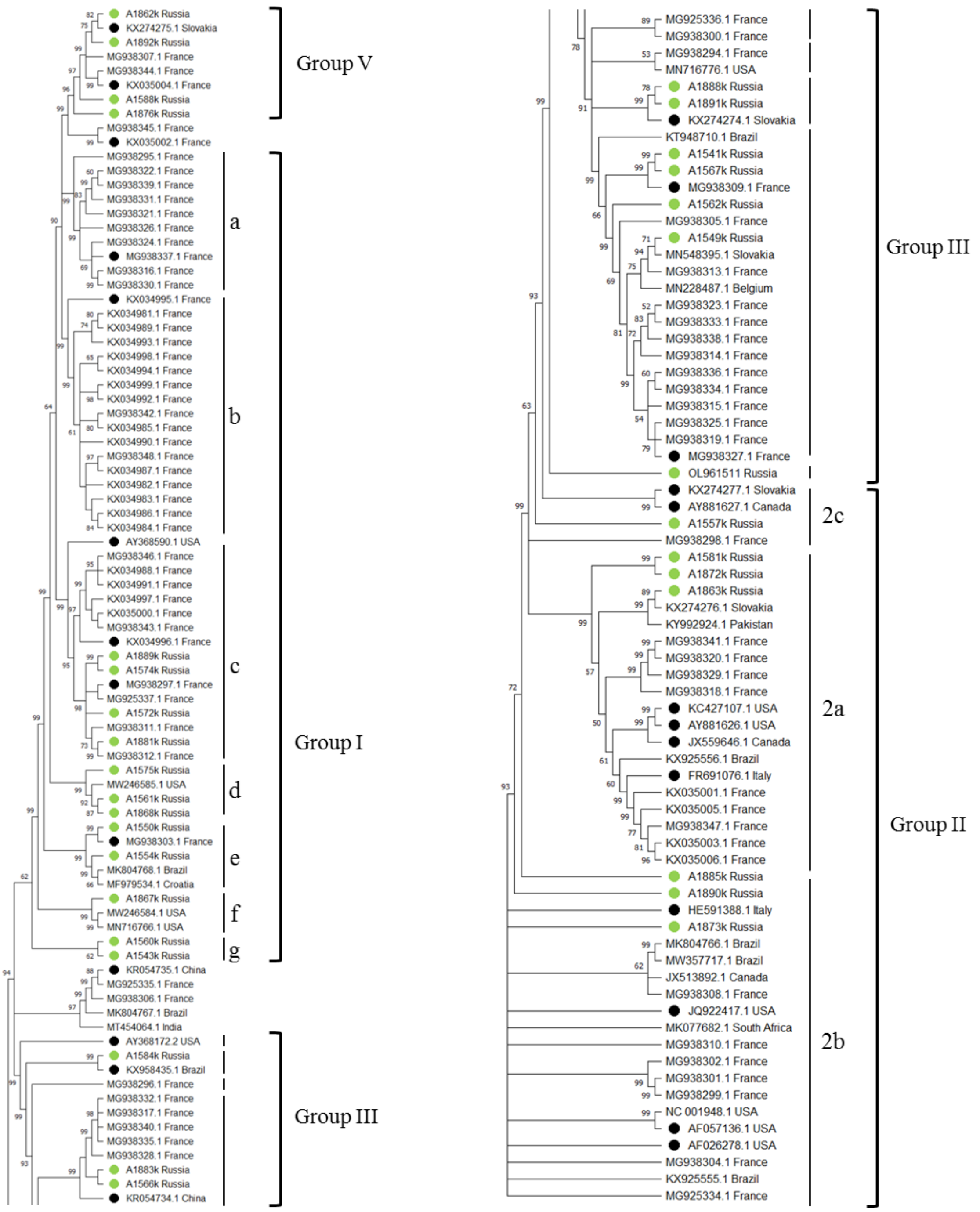

(a) **(b)**

Supplementary Figure S1 (a-b). Phylogenetic tree showing the distribution of coat protein nucleotide sequences of Russian *Grapevine rupestris stem pitting-associated virus* (GRSPaV) isolates. Neighbor Joining tree shows the distribution of Russian GRSPaV coat protein nucleotide sequences (●) compared to isolates from the Genbank and reference sequences (●). Geographical origin is provided for each Russian isolate (in brackets). Bootstrap values >60% (1000 bootstrap replicates) are shown.

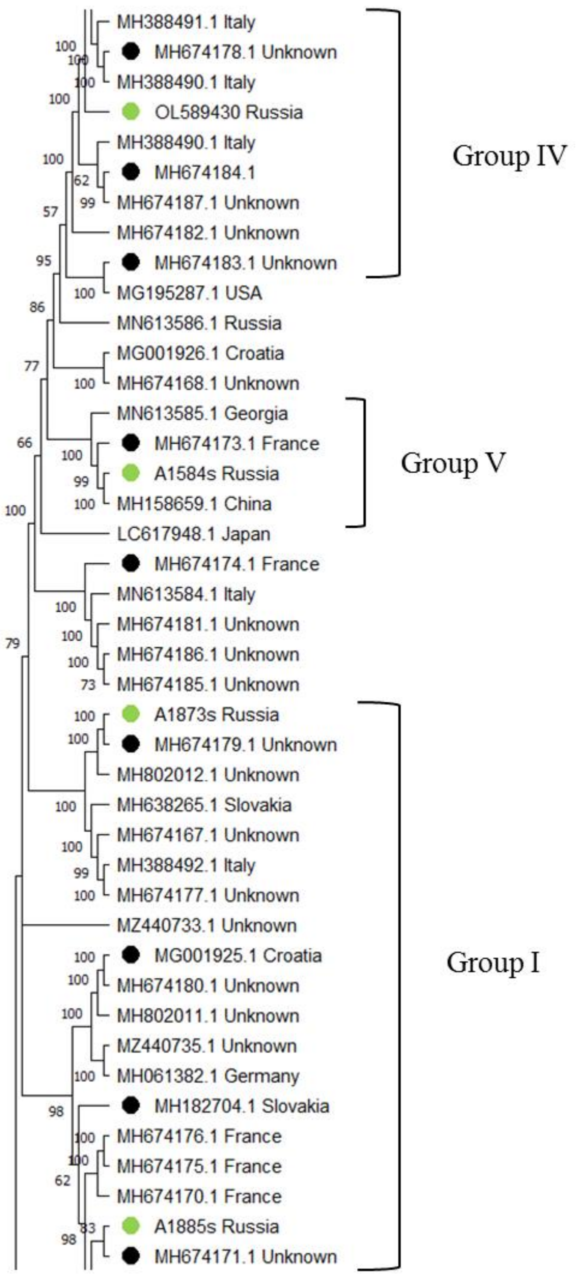

Supplementary Figure S2. Phylogenetic tree showing the distribution of complete genome nucleotide sequences of Russian *Grapevine virus T* (GVT) isolates. Neighbor Joining tree shows the distribution of Russian GVT complete genome nucleotide sequences (●) compared to isolates from the Genbank and reference sequences (●). Geographical origin is provided for each isolate. Bootstrap values >60% (1000 bootstrap replicates) are shown.

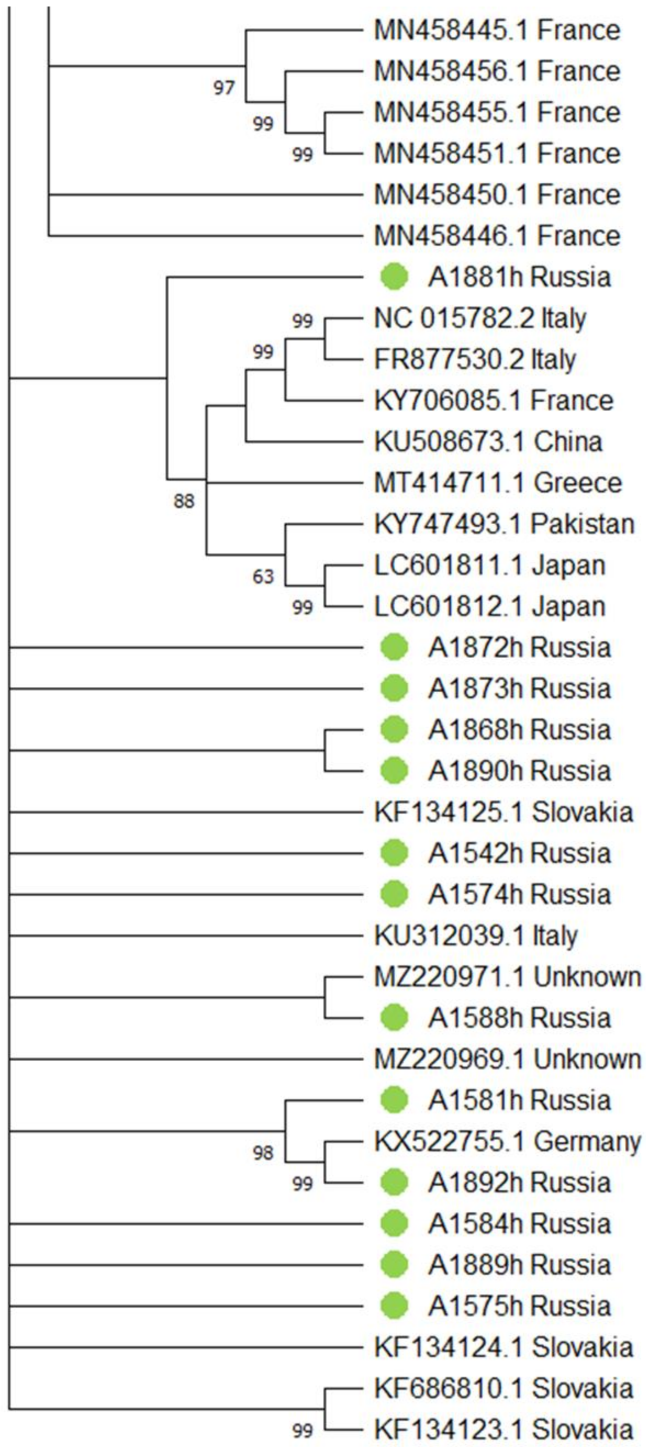

Supplementary Figure S3. Phylogenetic tree showing the distribution of complete genome nucleotide sequences of Russian *Grapevine Pinot gris virus* (GPGV) isolates. Neighbor Joining tree shows the distribution of Russian GPGV complete genome nucleotide sequences (●) compared to isolates. Geographical origin is provided for each isolate. Bootstrap values >60% (1000 bootstrap replicates) are shown.

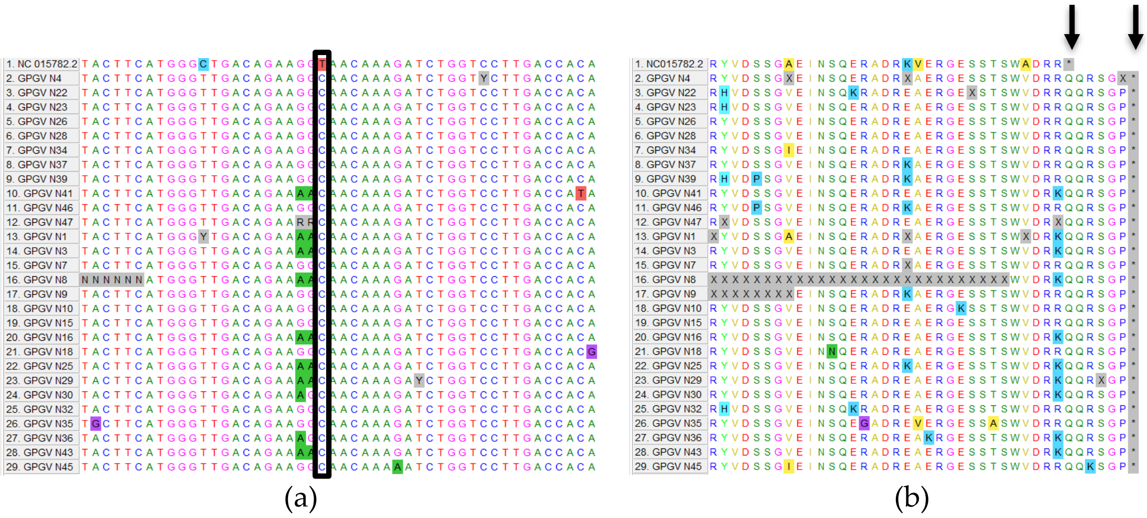

Supplementary Figure S4 (a, b). Alignment of (**a**) MP/CP nucleotide sequences; (**b**) movement proteins (MP) of Russian *Grapevine Pinot gris* virus (GPGV) isolates. 6685 T/C polymorphisms are marked in boxes. The stop codons are indicated by gray stars.

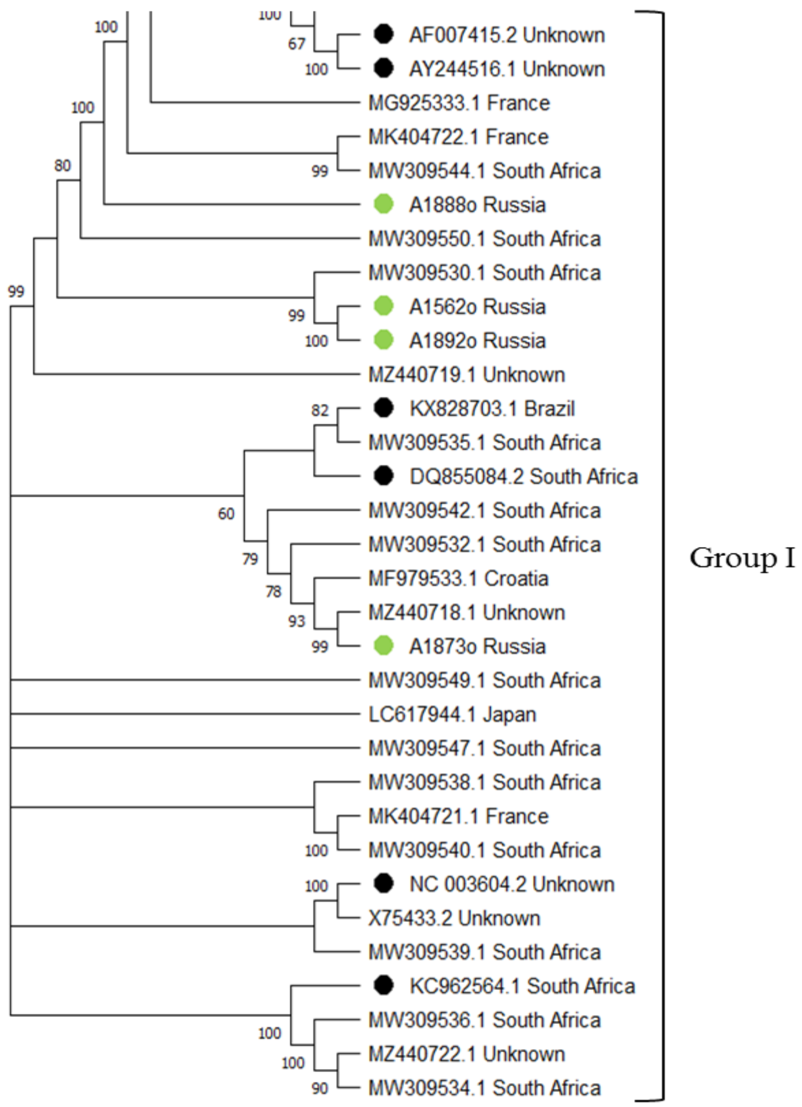

Supplementary Figure S5. Phylogenetic tree showing the distribution of complete genome nucleotide sequences of Russian *Grapevine virus A* (GVA) isolates. Neighbor Joining tree shows the distribution of Russian GVA complete genome nucleotide sequences (●) compared to isolates from the Genbank and reference sequences (●). Geographical origin is provided for each isolate. Bootstrap values >60% (1000 bootstrap replicates) are shown.

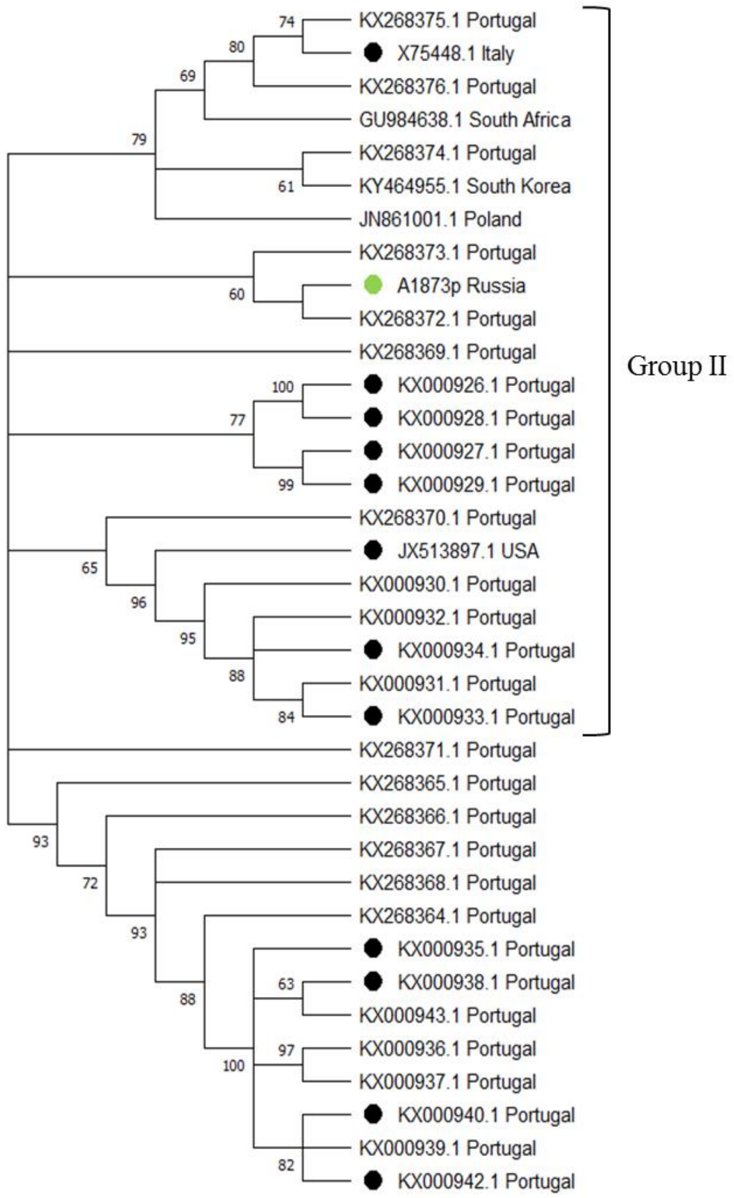

Supplementary Figure S6. Phylogenetic tree showing the distribution of coat protein nucleotide sequences of Russian *Grapevine virus B* (GVB) isolate. Neighbor Joining tree shows the distribution of Russian GVB coat protein nucleotide sequences (●) compared to isolates from the Genbank and reference sequences (●). Geographical origin is provided for each Russian isolate (in brackets). Bootstrap values >60% (1000 bootstrap replicates) are shown.

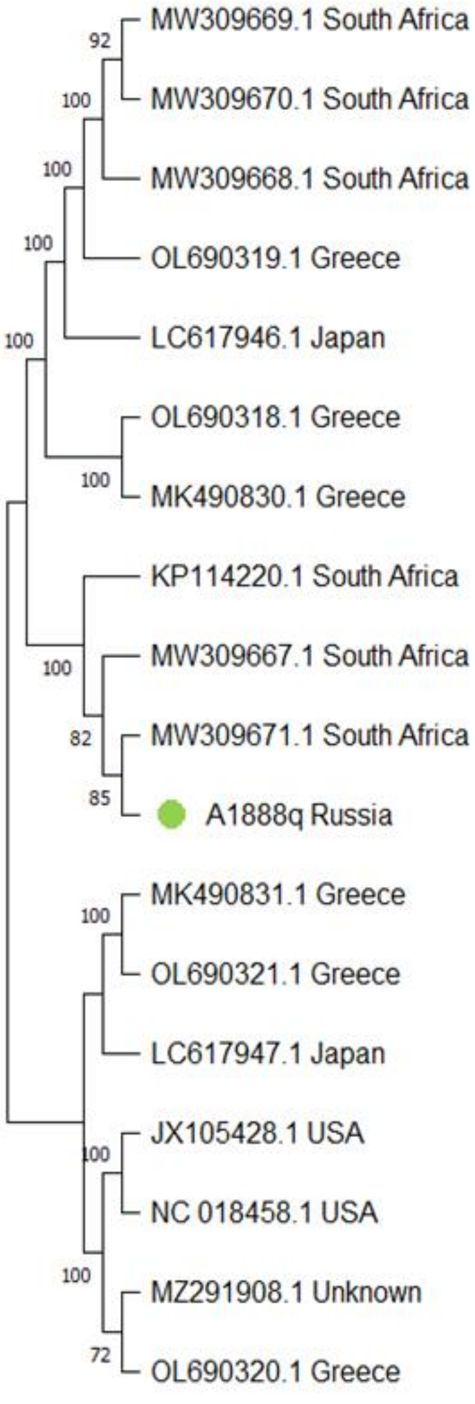

Supplementary Figure S7. Phylogenetic tree showing the distribution of complete genome nucleotide sequences of Russian *Grapevine virus F* (GVF) isolate. Neighbor Joining tree shows the distribution of Russian GVF complete genome nucleotide sequences (●) compared to isolates. Geographical origin is provided for each isolate. Bootstrap values >60% (1000 bootstrap replicates) are shown.

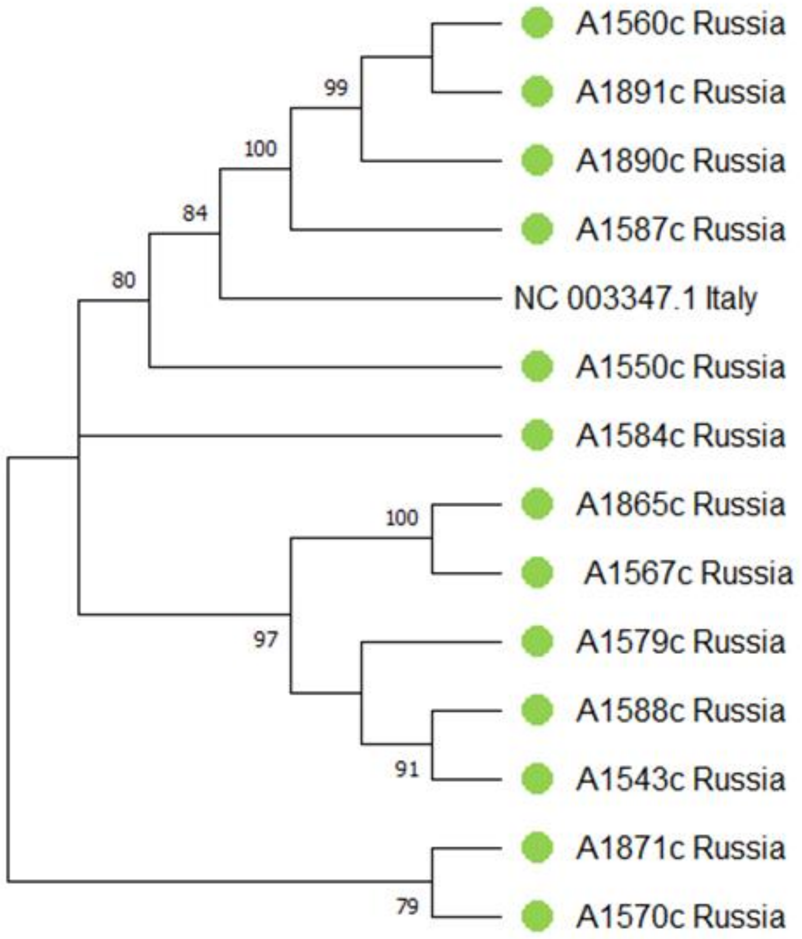

Supplementary Figure S8. Phylogenetic tree showing the distribution of complete genome nucleotide sequences of Russian *Grapevine fleck virus* (GFkV) isolates. Neighbor Joining tree shows the distribution of Russian GFkV complete genome nucleotide sequences (●) compared to isolates. Geographical origin is provided for each isolate. Bootstrap values >60% (1000 bootstrap replicates) are shown.

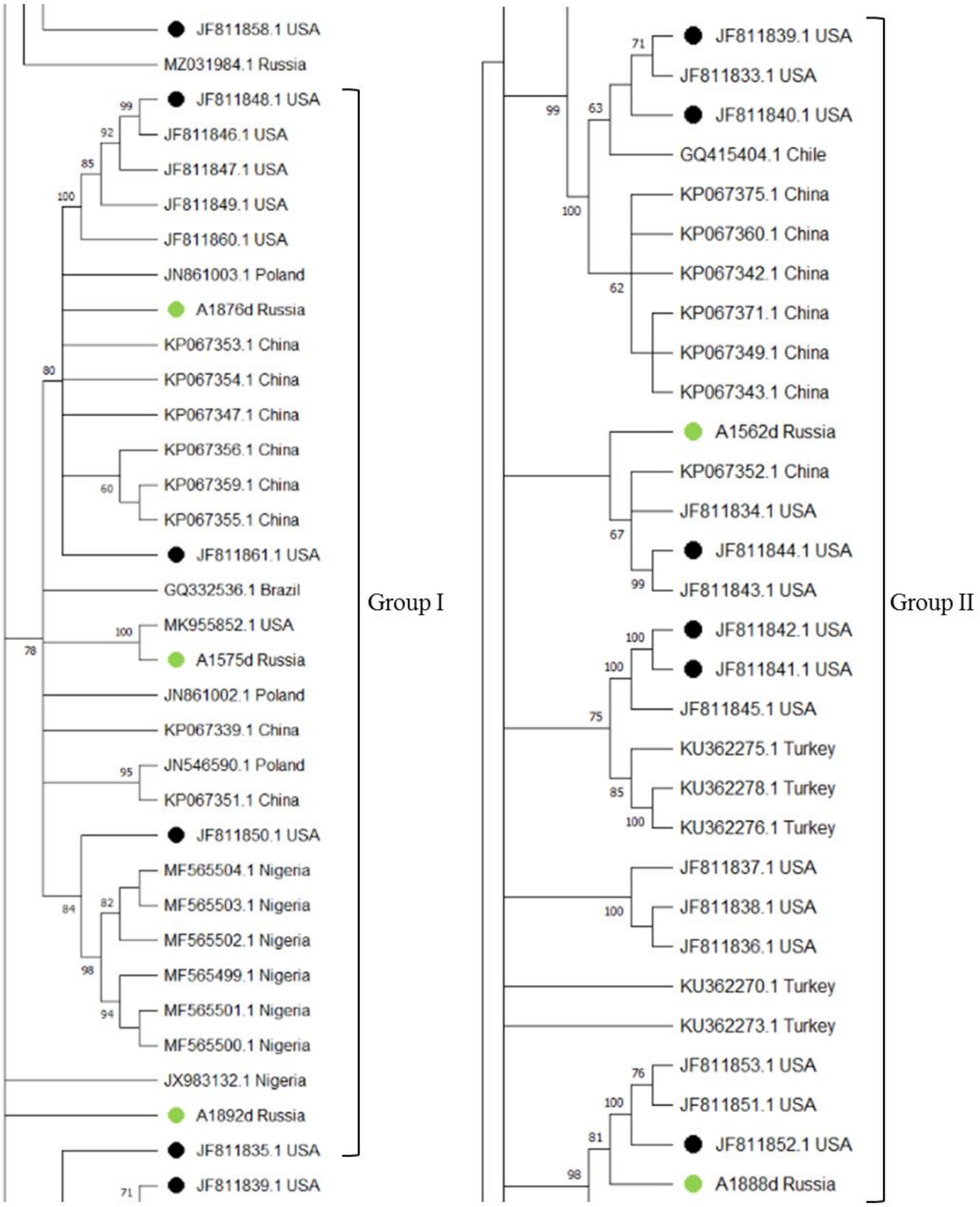

(a) **(b)**

Supplementary Figure S9 (a, b). Phylogenetic tree showing the distribution of coat protein nucleotide sequences of Russian *Grapevine leafroll-associated virus 1* (GLRaV-1) isolates. Neighbor Joining tree shows the distribution of Russian GLRaV-1 coat protein nucleotide sequences (●) compared to isolates from the Genbank and reference sequences (●). Geographical origin is provided for each Russian isolate (in brackets). Bootstrap values >60% (1000 bootstrap replicates) are shown.

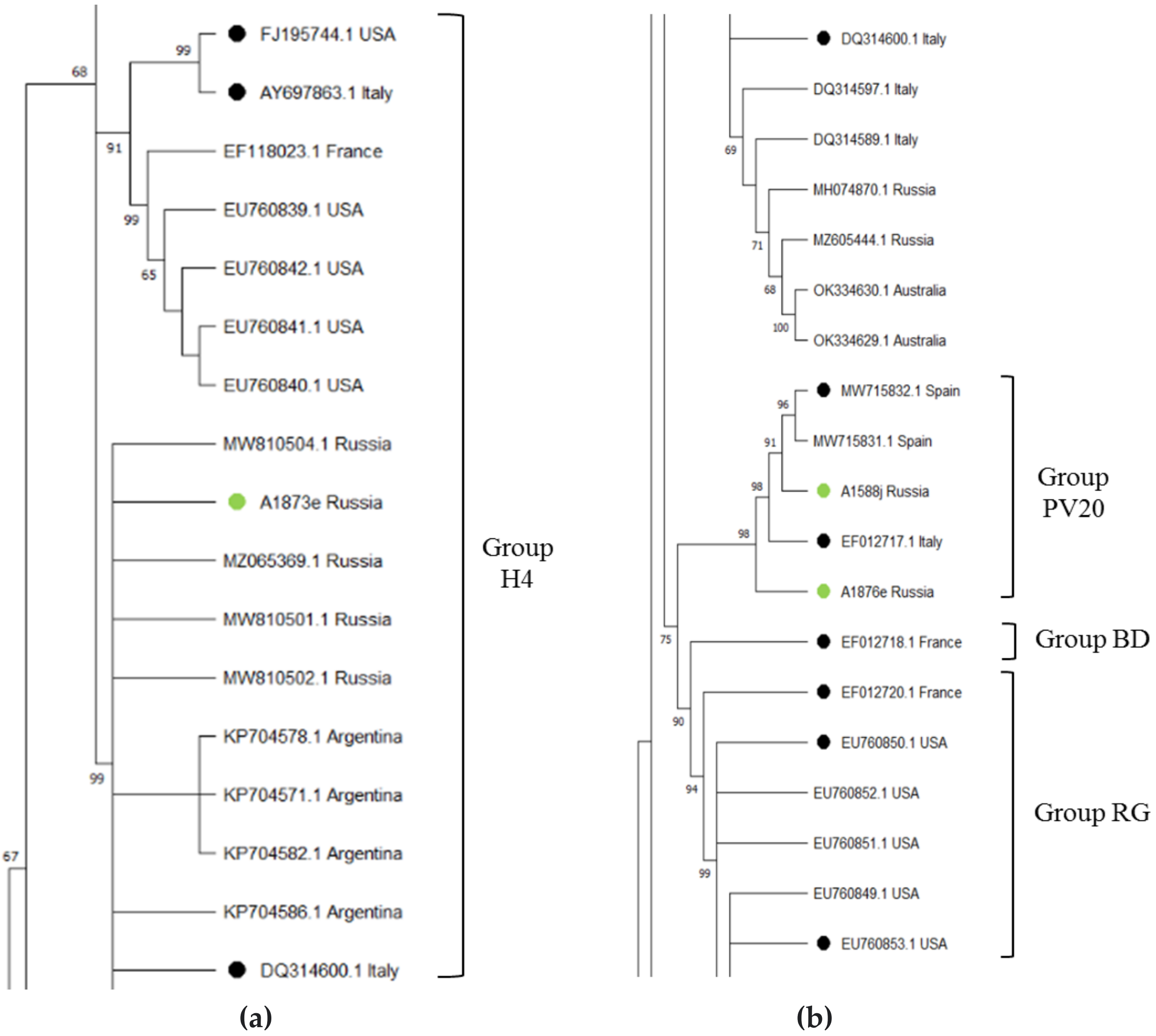
FJ195744.1 USA
AY697863.1 Italy
EF118023.1 France
EU760839.1 USA
EU760842.1 USA
EU760841.1 USA
EU760840.1 USA
MW810504.1 Russia
A1873e Russia
MZ065369.1 Russia
MW810501.1 Russia
MW810502.1 Russia
KP704578.1 Argentina
KP704571.1 Argentina
KP704582.1 Argentina
KP704586.1 Argentina
DQ314600.1 Italy
Group H4
(a)
DQ314600.1 Italy
DQ314597.1 Italy
DQ314589.1 Italy
MH074870.1 Russia
MZ605444.1 Russia
OK334630.1 Australia
OK334629.1 Australia
MW715832.1 Spain
MW715831.1 Spain
A1588j Russia
EF012717.1 Italy
A1876e Russia
Group PV20
EF012718.1 France
Group BD
EF012720.1 France
EU760850.1 USA
EU760852.1 USA
EU760851.1 USA
EU760849.1 USA
EU760853.1 USA
Group RG
(b)

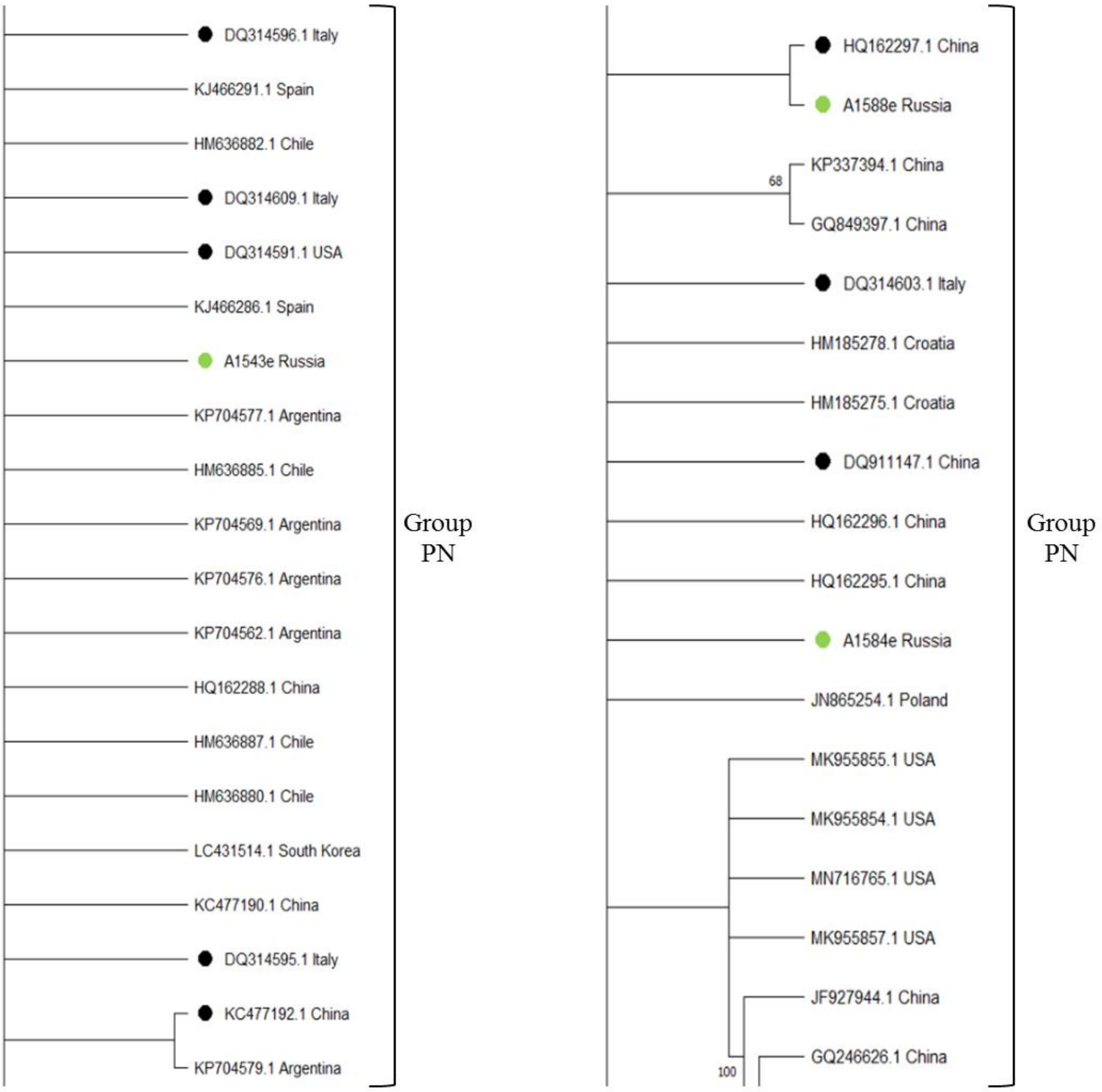

(c) **(d)**

Supplementary Figure S10 (a-d). Phylogenetic tree showing the distribution of coat protein nucleotide sequences of Russian *Grapevine leafroll-associated virus 2* (GLRaV-2) isolates. Neighbor Joining tree shows the distribution of Russian GLRaV-2 coat protein nucleotide sequences (●) compared to isolates from the Genbank and reference sequences (●). Geographical origin is provided for each Russian isolate (in brackets). Bootstrap values >60% (1000 bootstrap replicates) are shown.

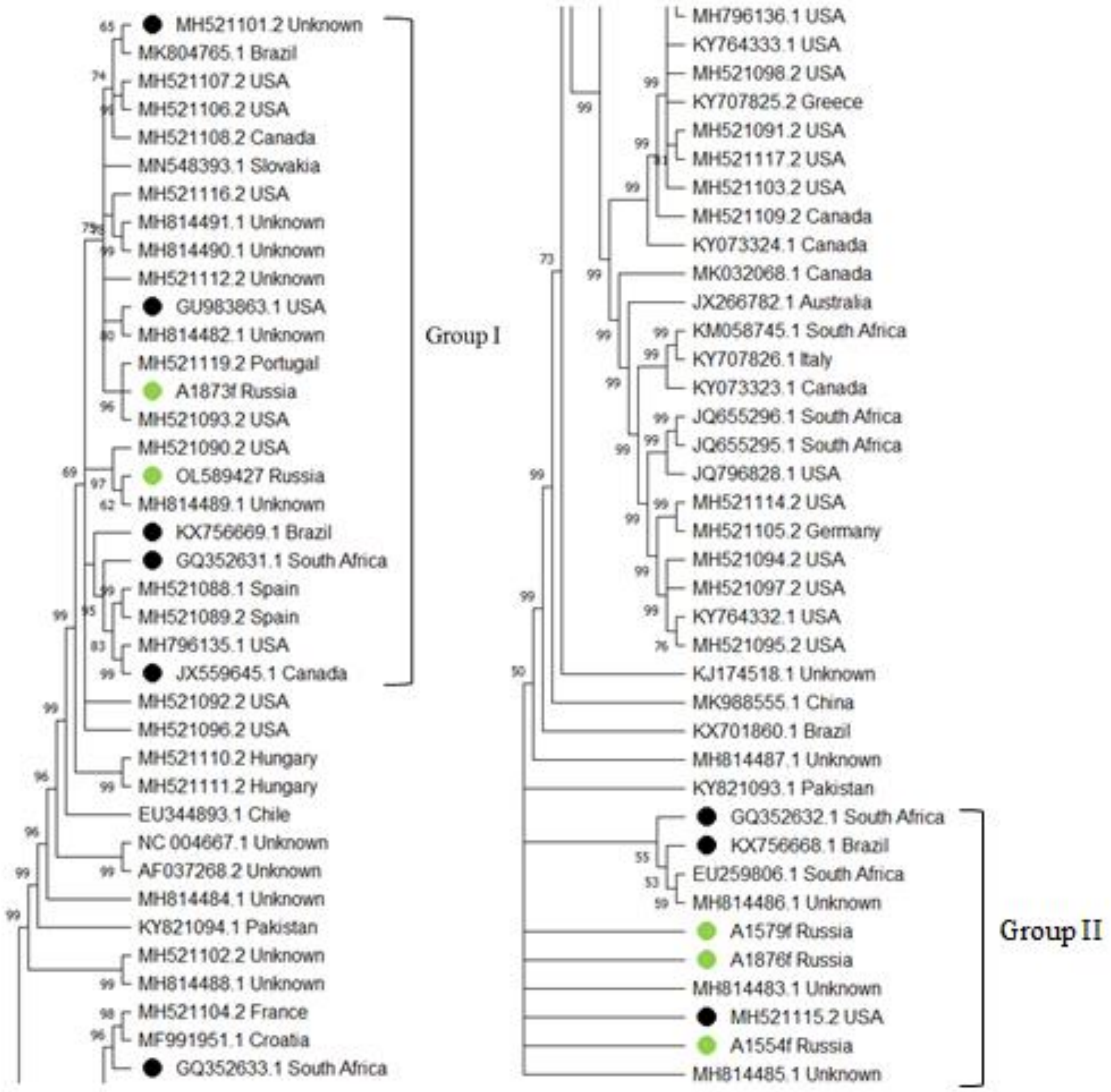

(a) **(b)**

Supplementary Figure S11 (a, b). Phylogenetic tree showing the distribution of complete genome nucleotide sequences of Russian *Grapevine leafroll-associated virus 3* (GLRaV-3) isolates. Neighbor Joining tree shows the distribution of Russian GLRaV-3 complete genome nucleotide sequences (●) compared to isolates from the Genbank and reference sequences (●). Geographical origin is provided for each isolate. Bootstrap values >60% (1000 bootstrap replicates) are shown.

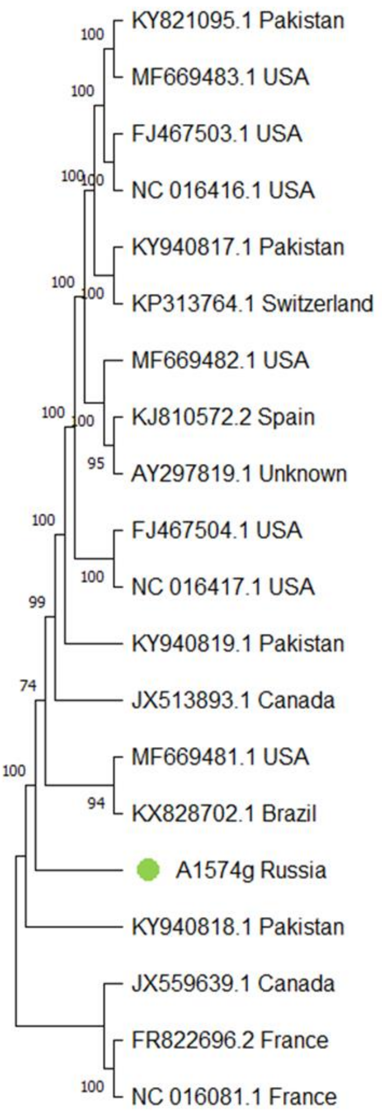

Supplementary Figure S12. Phylogenetic tree showing the distribution of complete genome nucleotide sequences of Russian *Grapevine leafroll-associated virus 4* (GLRaV-4 strain GLRaV-5) isolate. Neighbor Joining tree shows the distribution of Russian GLRaV-4 complete genome nucleotide sequences (●) compared to isolates. Geographical origin is provided for each isolate. Bootstrap values >60% (1000 bootstrap replicates) are shown.

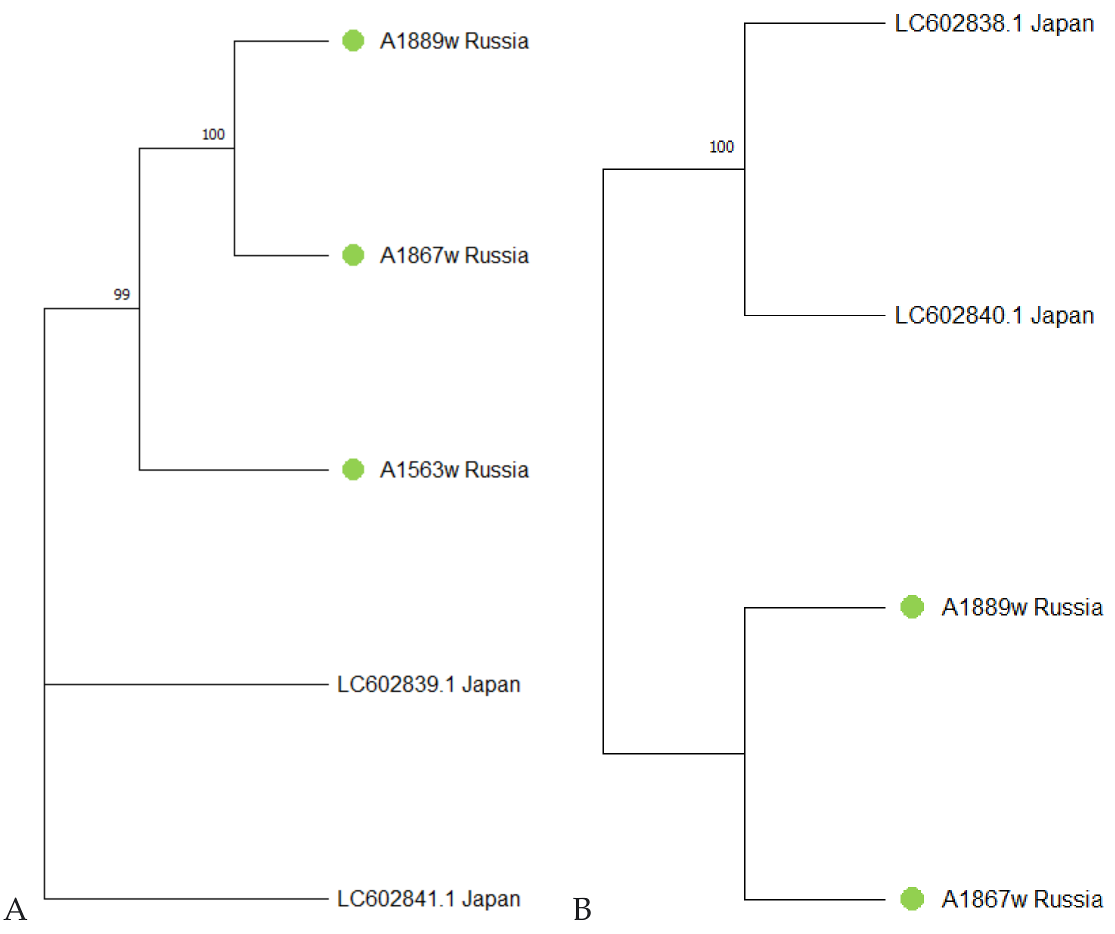

(a) **(b)**

Supplementary Figure S13 (a, b). Phylogenetic tree showing the distribution of complete genome nucleotide sequences (a) – coat protein (CP) and (b) – RNA-dependent RNA polymerase (RdRp) of Russian *Vitis cryptic virus* (VCV) isolates. Neighbor Joining tree shows the distribution of Russian VCV complete genome nucleotide sequences (●) compared to isolates. Geographical origin is provided for each isolate. Bootstrap values >60% (1000 bootstrap replicates) are shown.

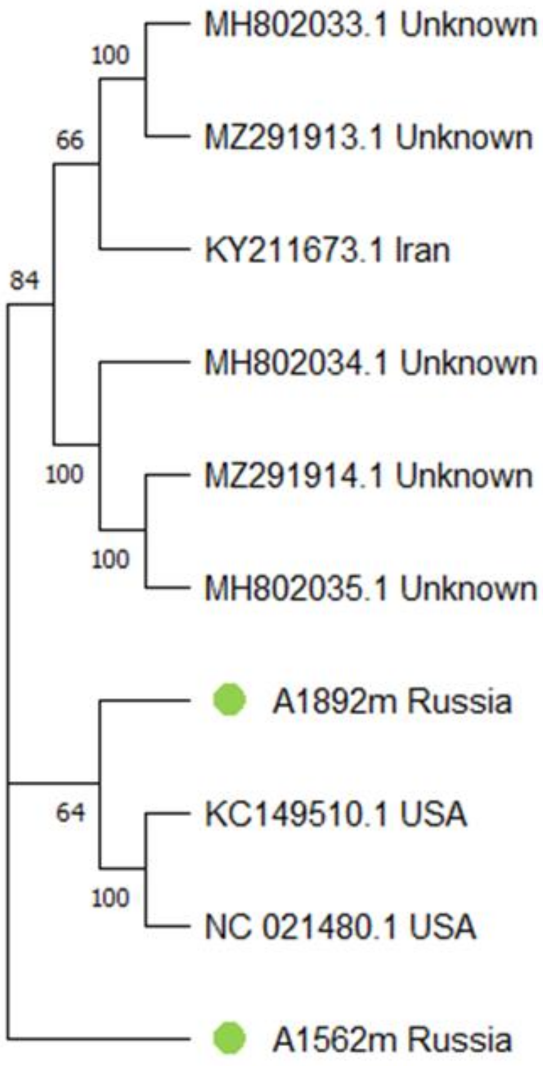

Supplementary Figure S14. Phylogenetic tree showing the distribution of complete genome nucleotide sequences of Russian *Grapevine satellite virus* (GV-Sat) isolates. Neighbor Joining tree shows the distribution of Russian GV-Sat complete genome nucleotide sequences (●) compared to isolates. Geographical origin is provided for each isolate. Bootstrap values >60% (1000 bootstrap replicates) are shown.

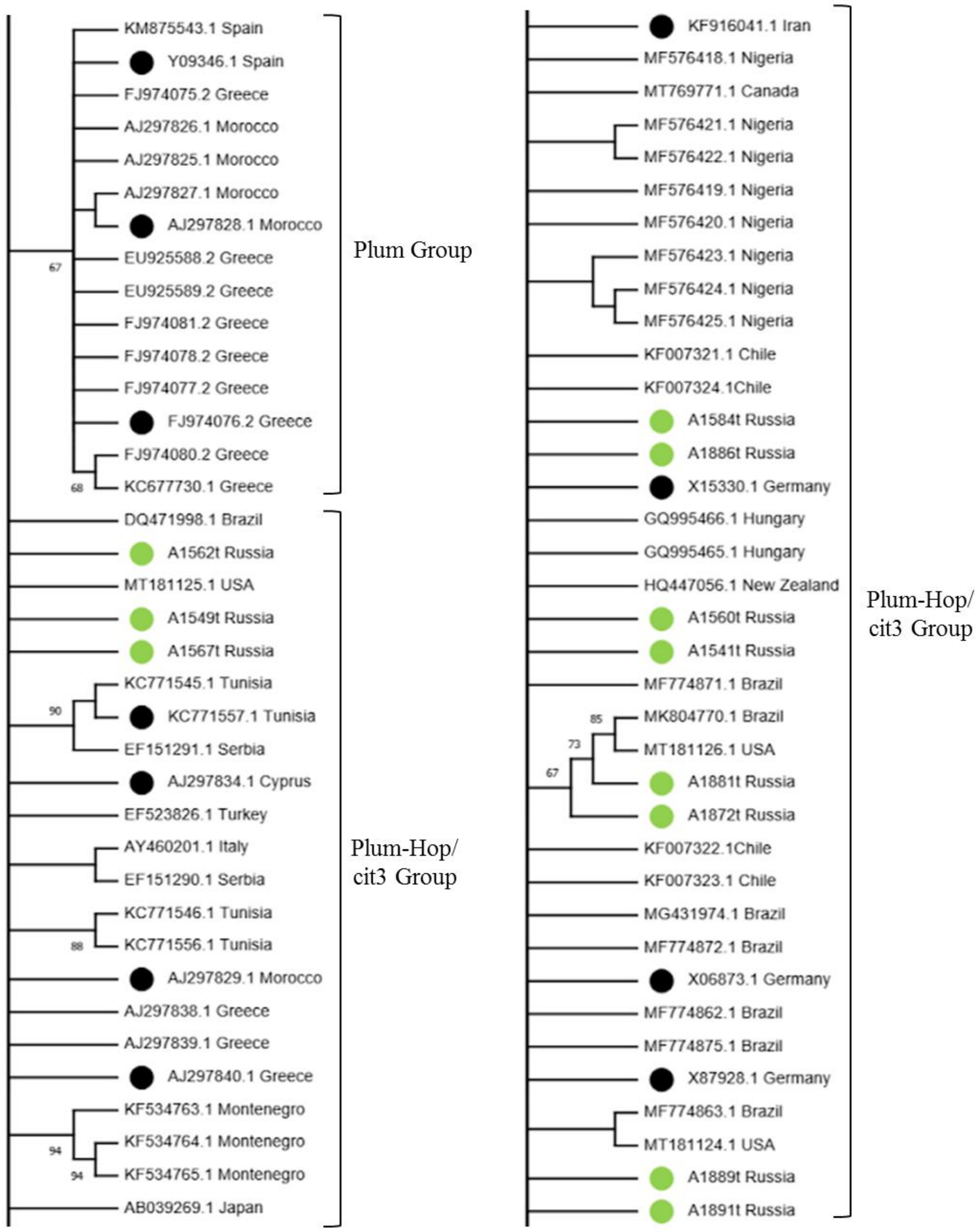
KM875543.1 Spain
Y09346.1 Spain
FJ974075.2 Greece
AJ297826.1 Morocco
AJ297825.1 Morocco
AJ297827.1 Morocco
AJ297828.1 Morocco
EU925588.2 Greece
EU925589.2 Greece
FJ974081.2 Greece
FJ974078.2 Greece
FJ974077.2 Greece
FJ974076.2 Greece
FJ974080.2 Greece
KC677730.1 Greece
67
68
Plum Group
DQ471998.1 Brazil
A1562t Russia
MT181125.1 USA
A1549t Russia
A1567t Russia
KC771545.1 Tunisia
KC771557.1 Tunisia
EF151291.1 Serbia
90
AJ297834.1 Cyprus
EF523826.1 Turkey
AY460201.1 Italy
EF151290.1 Serbia
KC771546.1 Tunisia
KC771556.1 Tunisia
88
AJ297829.1 Morocco
AJ297838.1 Greece
AJ297839.1 Greece
AJ297840.1 Greece
KF534763.1 Montenegro
KF534764.1 Montenegro
KF534765.1 Montenegro
94
94
AB039269.1 Japan
Plum-Hop/
cit3 Group
KF916041.1 Iran
MF576418.1 Nigeria
MT769771.1 Canada
MF576421.1 Nigeria
MF576422.1 Nigeria
MF576419.1 Nigeria
MF576420.1 Nigeria
MF576423.1 Nigeria
MF576424.1 Nigeria
MF576425.1 Nigeria
KF007321.1 Chile
KF007324.1Chile
A1584t Russia
A1886t Russia
X15330.1 Germany
GQ995466.1 Hungary
GQ995465.1 Hungary
HQ447056.1 New Zealand
A1560t Russia
A1541t Russia
MF774871.1 Brazil
MK804770.1 Brazil
MT181126.1 USA
A1881t Russia
A1872t Russia
85
73
67
KF007322.1Chile
KF007323.1 Chile
MG431974.1 Brazil
MF774872.1 Brazil
X06873.1 Germany
MF774862.1 Brazil
MF774875.1 Brazil
X87928.1 Germany
MF774863.1 Brazil
MT181124.1 USA
A1889t Russia
A1891t Russia
Plum-Hop/
cit3 Group

(a) **(b)**

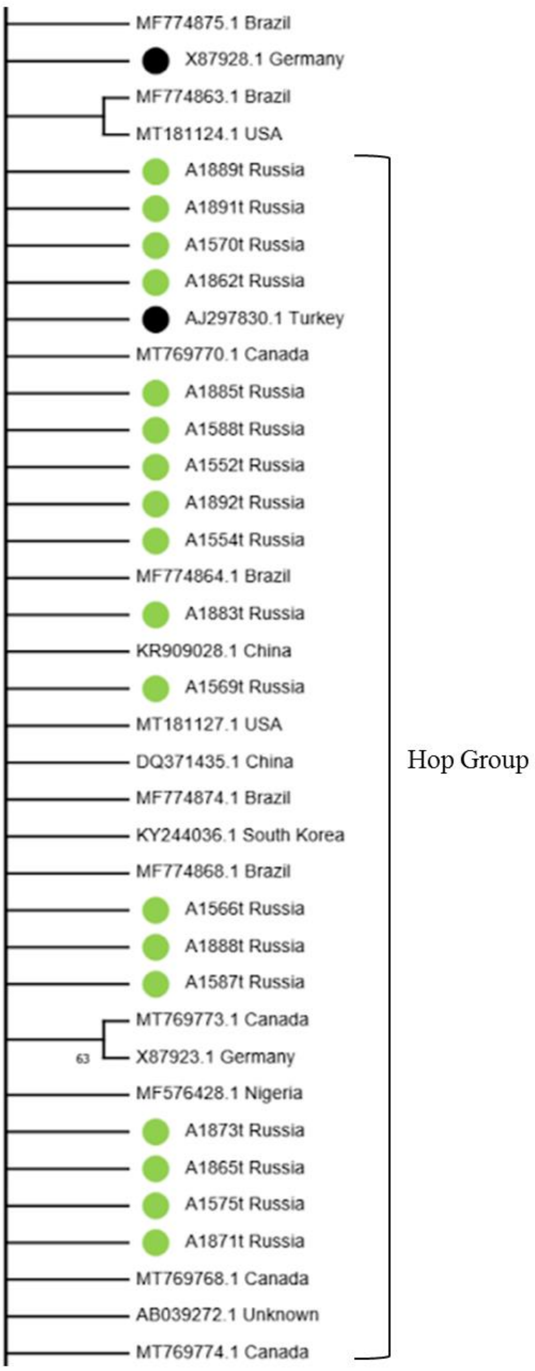
MF774875.1 Brazil
X87928.1 Germany
MF774863.1 Brazil
MT181124.1 USA
A1889t Russia
A1891t Russia
A1570t Russia
A1862t Russia
AJ297830.1 Turkey
MT769770.1 Canada
A1885t Russia
A1588t Russia
A1552t Russia
A1892t Russia
A1554t Russia
MF774864.1 Brazil
A1883t Russia
KR909028.1 China
A1569t Russia
MT181127.1 USA
DQ371435.1 China
MF774874.1 Brazil
KY244036.1 South Korea
MF774868.1 Brazil
A1566t Russia
A1888t Russia
A1587t Russia
MT769773.1 Canada
63
X87923.1 Germany
MF576428.1 Nigeria
A1873t Russia
A1865t Russia
A1575t Russia
A1871t Russia
MT769768.1 Canada
AB039272.1 Unknown
MT769774.1 Canada
Hop Group

(c)

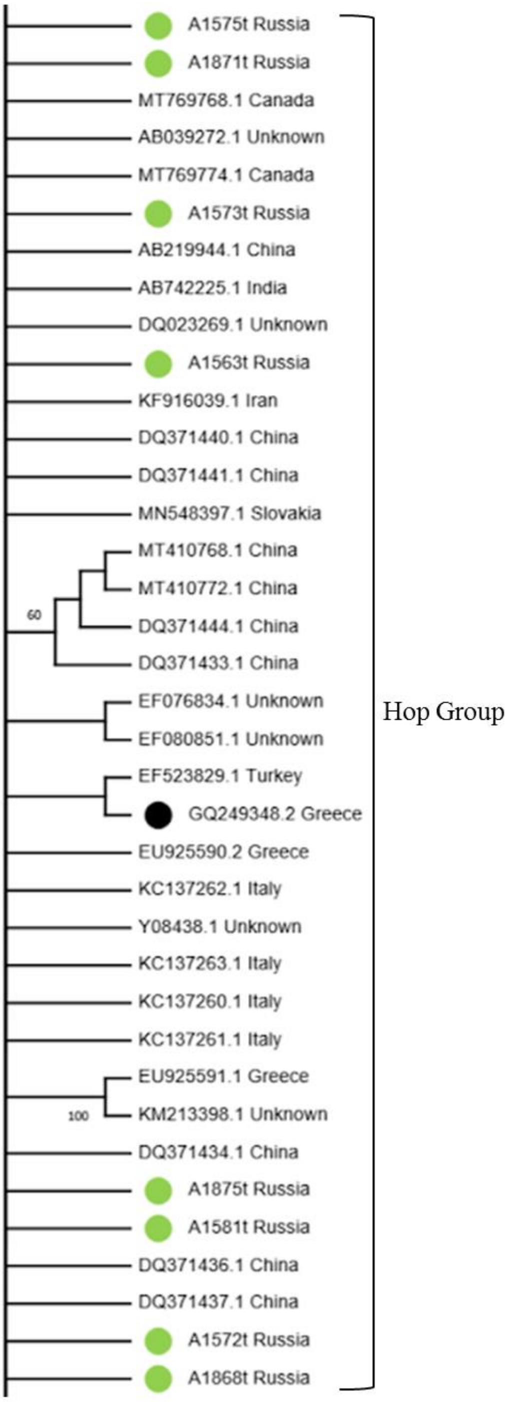
A1575t Russia
A1871t Russia
MT769768.1 Canada
AB039272.1 Unknown
MT769774.1 Canada
A1573t Russia
AB219944.1 China
AB742225.1 India
DQ023269.1 Unknown
A1563t Russia
KF916039.1 Iran
DQ371440.1 China
DQ371441.1 China
MN548397.1 Slovakia
MT410768.1 China
MT410772.1 China
60
DQ371444.1 China
DQ371433.1 China
EF076834.1 Unknown
EF080851.1 Unknown
EF523829.1 Turkey
GQ249348.2 Greece
EU925590.2 Greece
KC137262.1 Italy
Y08438.1 Unknown
KC137263.1 Italy
KC137260.1 Italy
KC137261.1 Italy
EU925591.1 Greece
100
KM213398.1 Unknown
DQ371434.1 China
A1875t Russia
A1581t Russia
DQ371436.1 China
DQ371437.1 China
A1572t Russia
A1868t Russia
Hop Group

(d)

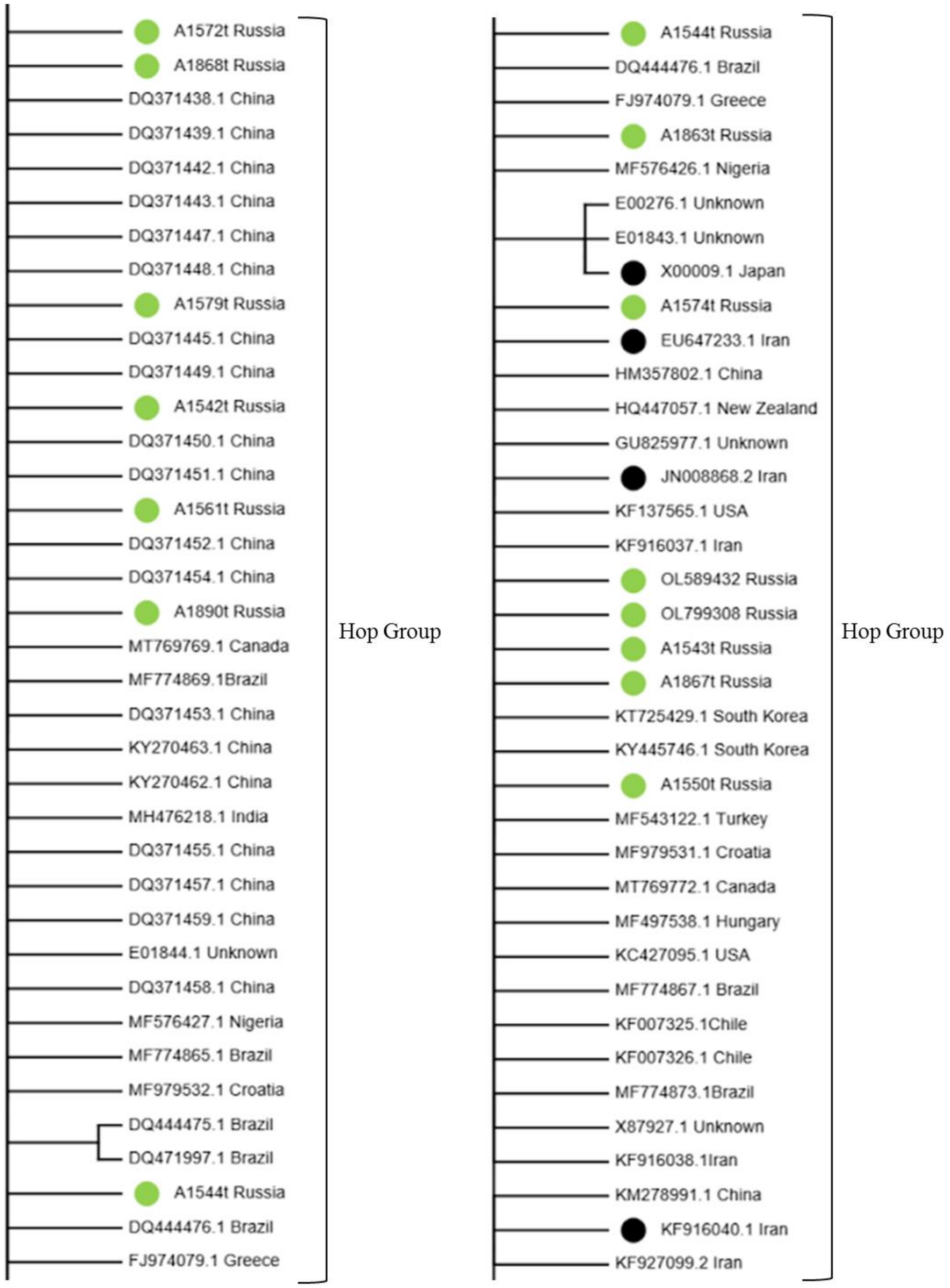

(e) **(f)**

Supplementary Figure S15 (a-f). Phylogenetic tree showing the distribution of complete genome nucleotide sequences of Russian *Hop stunt viroid* (HSVd) isolates. Neighbor Joining tree shows the distribution of Russian HSVd complete genome nucleotide sequences (●) compared to isolates from the Genbank and reference sequences (●). Geographical origin is provided for each isolate. Bootstrap values >60% (1000 bootstrap replicates) are shown.

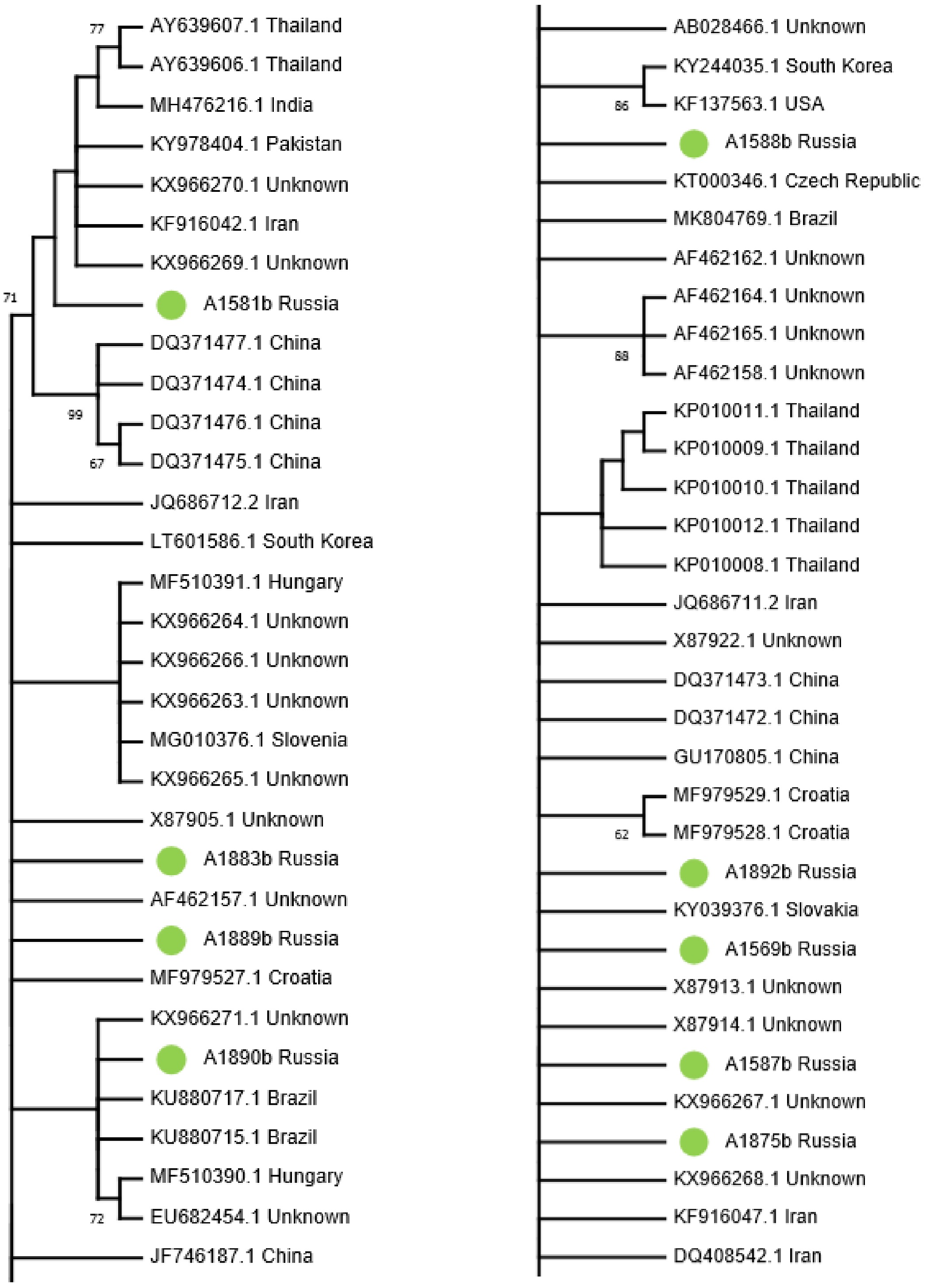

AY639607.1 Thailand
AY639606.1 Thailand
MH476216.1 India
KY978404.1 Pakistan
KX966270.1 Unknown
KF916042.1 Iran
KX966269.1 Unknown
A1581b Russia
DQ371477.1 China
DQ371474.1 China
DQ371476.1 China
DQ371475.1 China
JQ686712.2 Iran
LT601586.1 South Korea
MF510391.1 Hungary
KX966264.1 Unknown
KX966266.1 Unknown
KX966263.1 Unknown
MG010376.1 Slovenia
KX966265.1 Unknown
X87905.1 Unknown
A1883b Russia
AF462157.1 Unknown
A1889b Russia
MF979527.1 Croatia
KX966271.1 Unknown
A1890b Russia
KU880717.1 Brazil
KU880715.1 Brazil
MF510390.1 Hungary
EU682454.1 Unknown
JF746187.1 China
77
71
99
67
72
AB028466.1 Unknown
KY244035.1 South Korea
KF137563.1 USA
A1588b Russia
KT000346.1 Czech Republic
MK804769.1 Brazil
AF462162.1 Unknown
AF462164.1 Unknown
AF462165.1 Unknown
AF462158.1 Unknown
KP010011.1 Thailand
KP010009.1 Thailand
KP010010.1 Thailand
KP010012.1 Thailand
KP010008.1 Thailand
JQ686711.2 Iran
X87922.1 Unknown
DQ371473.1 China
DQ371472.1 China
GU170805.1 China
MF979529.1 Croatia
MF979528.1 Croatia
A1892b Russia
KY039376.1 Slovakia
A1569b Russia
X87913.1 Unknown
X87914.1 Unknown
A1587b Russia
KX966267.1 Unknown
A1875b Russia
KX966268.1 Unknown
KF916047.1 Iran
DQ408542.1 Iran
86
88
62

(a)

(b)

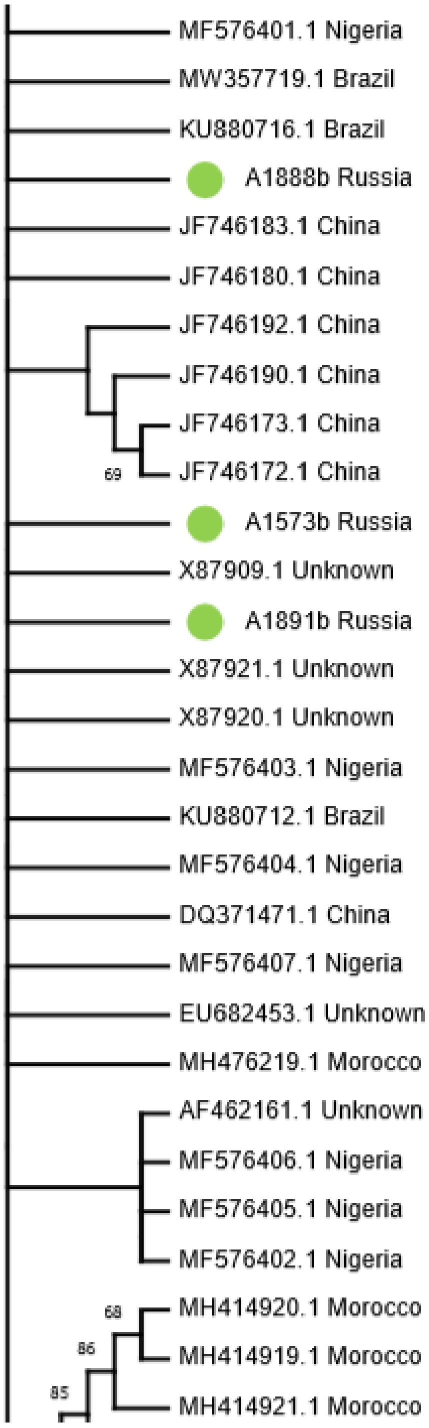
MF576401.1 Nigeria
MW357719.1 Brazil
KU880716.1 Brazil
A1888b Russia
JF746183.1 China
JF746180.1 China
JF746192.1 China
JF746190.1 China
JF746173.1 China
69
JF746172.1 China
A1573b Russia
X87909.1 Unknown
A1891b Russia
X87921.1 Unknown
X87920.1 Unknown
MF576403.1 Nigeria
KU880712.1 Brazil
MF576404.1 Nigeria
DQ371471.1 China
MF576407.1 Nigeria
EU682453.1 Unknown
MH476219.1 Morocco
AF462161.1 Unknown
MF576406.1 Nigeria
MF576405.1 Nigeria
MF576402.1 Nigeria
68
MH414920.1 Morocco
86
MH414919.1 Morocco
85
MH414921.1 Morocco

(c)

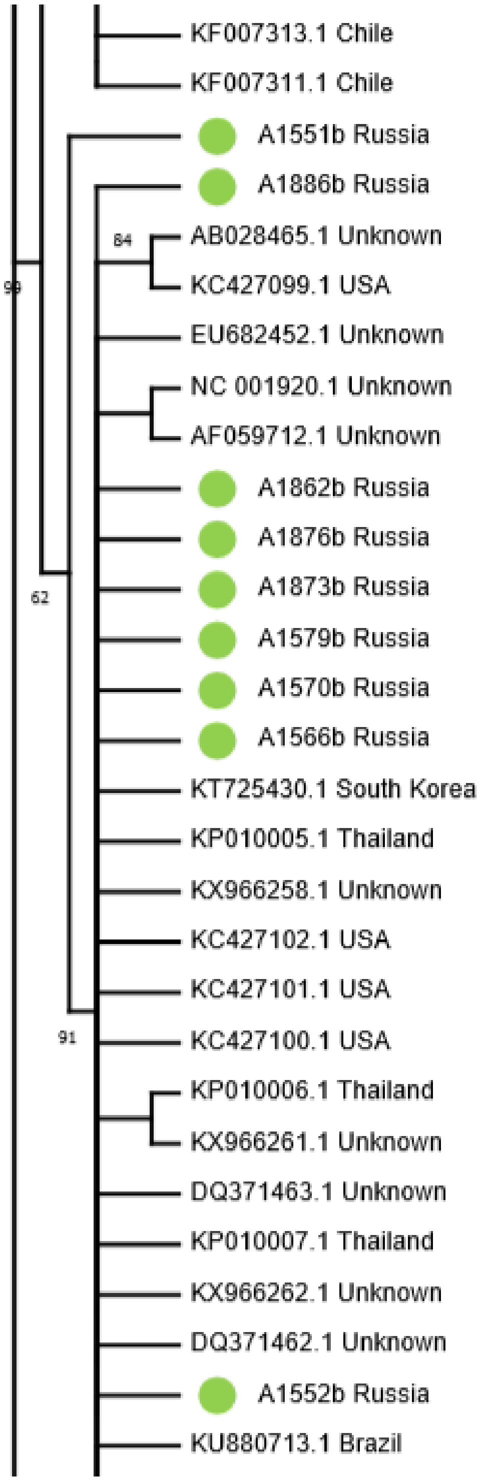
KF007313.1 Chile
KF007311.1 Chile
A1551b Russia
A1886b Russia
84
AB028465.1 Unknown
KC427099.1 USA
EU682452.1 Unknown
NC 001920.1 Unknown
AF059712.1 Unknown
A1862b Russia
A1876b Russia
A1873b Russia
62
A1579b Russia
A1570b Russia
A1566b Russia
KT725430.1 South Korea
KP010005.1 Thailand
KX966258.1 Unknown
KC427102.1 USA
KC427101.1 USA
91
KC427100.1 USA
KP010006.1 Thailand
KX966261.1 Unknown
DQ371463.1 Unknown
KP010007.1 Thailand
KX966262.1 Unknown
DQ371462.1 Unknown
A1552b Russia
KU880713.1 Brazil

(d)

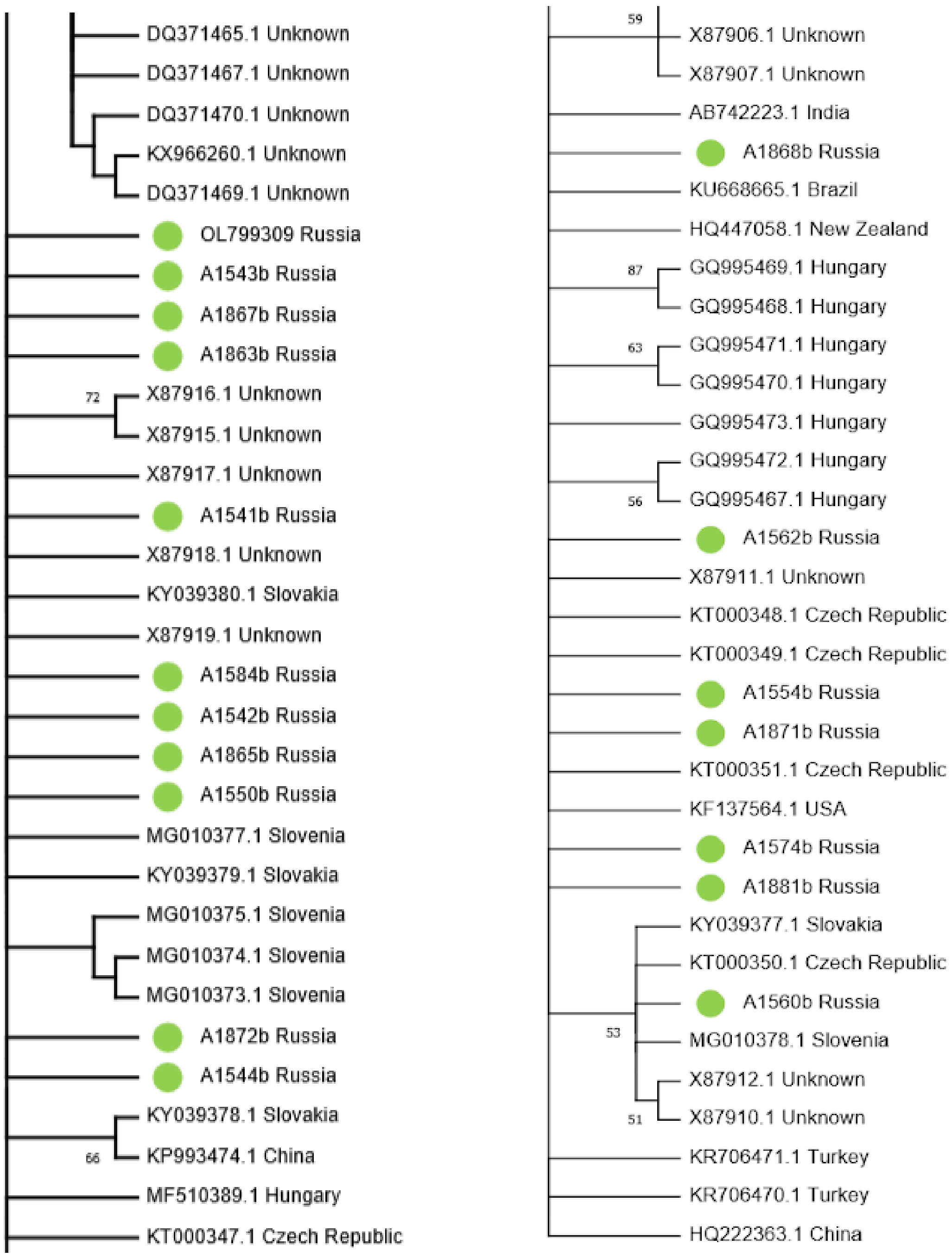

(e) **(f)**

Supplementary Figure S16 (a-f). Phylogenetic tree showing the distribution of complete genome nucleotide sequences of Russian *Grapevine yellow speckle viroid 1* (GYSVd-1) isolates. Neighbor Joining tree shows the distribution of Russian GYSVd-1 complete genome nucleotide sequences (●) compared to isolates. Geographical origin is provided for each isolate. Bootstrap values >60% (1000 bootstrap replicates) are shown.

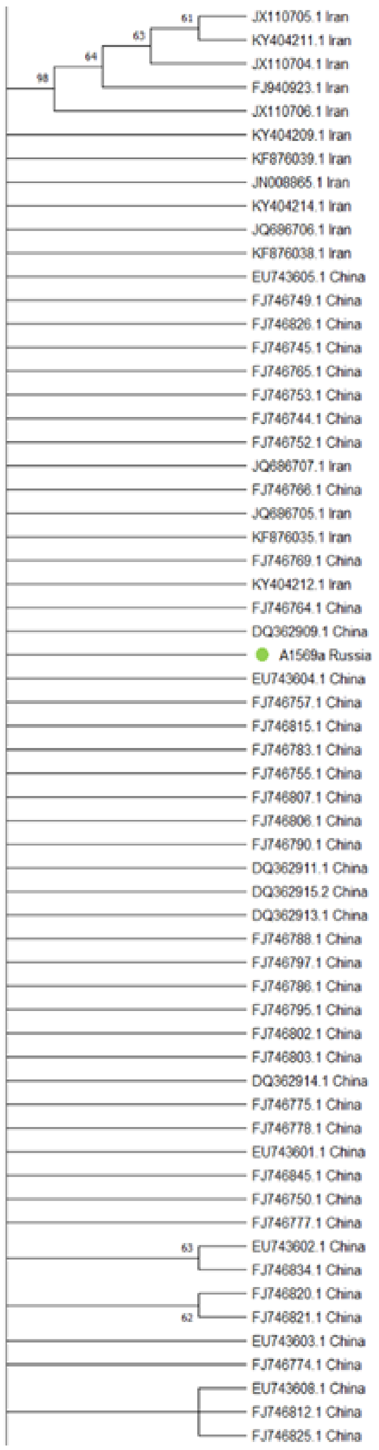

Supplementary Figure S17. Phylogenetic tree showing the distribution of complete genome nucleotide sequences of Russian *Australian grapevine viroid* (AGVd) isolate. Neighbor Joining tree shows the distribution of Russian AGVd complete genome nucleotide sequences (●) compared to isolates. Geographical origin is provided for each isolate. Bootstrap values >60% (1000 bootstrap replicates) are shown.
